# Study protocol: How does cognitive flexibility relate to other executive functions and learning in healthy young adults?

**DOI:** 10.1371/journal.pone.0286208

**Published:** 2023-07-20

**Authors:** Ke Tong, Yuan Ni Chan, Xiaoqin Cheng, Bobby Cheon, Michelle Ellefson, Restria Fauziana, Shengchuang Feng, Nastassja Fischer, Balázs Gulyás, Natalie Hoo, David Hung, Kastoori Kalaivanan, Christelle Langley, Kean Mun Lee, Li Ling Lee, Timothy Lee, Irene Melani, Nadhilla Melia, Jia Ying Pei, Lisha Raghani, Yoke Loo Sam, Peter Seow, John Suckling, Yan Fen Tan, Chew Lee Teo, Ryutaro Uchiyama, Hui Shan Yap, Georgios Christopoulos, Henriette Hendriks, Annabel Chen, Trevor Robbins, Barbara Sahakian, Zoe Kourtzi, Victoria Leong, CLIC Phase 1 Consortium

**Affiliations:** 1 Nanyang Technological University, Singapore, Singapore; 2 National Institutes of Health, Bethesda, Maryland, United States of America; 3 University of Cambridge, Cambridge, United Kingdom; 4 National Institute of Education, Singapore, Singapore; Public Library of Science, UNITED STATES

## Abstract

**Background:**

Cognitive flexibility (CF) enables individuals to readily shift from one concept or mode of practice/thoughts to another in response to changes in the environment and feedback, making CF vital to optimise success in obtaining goals. However, how CF relates to other executive functions (e.g., working memory, response inhibition), mental abilities (e.g., creativity, literacy, numeracy, intelligence, structure learning), and social factors (e.g., multilingualism, tolerance of uncertainty, perceived social support, social decision-making) is less well understood. The current study aims to (1) establish the construct validity of CF in relation to other executive function skills and intelligence, and (2) elucidate specific relationships between CF, structure learning, creativity, career decision making and planning, and other life skills.

**Methods:**

This study will recruit up to 400 healthy Singaporean young adults (age 18–30) to complete a wide range of cognitive tasks and social questionnaires/tasks. The richness of the task/questionnaire battery and within-participant administration enables us to use computational modelling and structural equation modelling to examine connections between the latent constructs of interest.

**Significance and Impact:**

The current study is the first systematic investigation into the construct validity of CF and its interrelationship with other important cognitive skills such as learning and creativity, within an Asian context. The study will further explore the concept of CF as a non-unitary construct, a novel theoretical proposition in the field. The inclusion of a structure learning paradigm is intended to inform future development of a novel intervention paradigm to enhance CF. Finally, the results of the study will be useful for informing classroom pedagogy and the design of lifelong learning policies and curricula, as part of the wider remit of the Cambridge-NTU Centre for Lifelong Learning and Individualised Cognition (CLIC).

## Introduction

As technology and globalisation are changing the nature of labour markets and increasing the demand for high skills levels, the need for individuals to be capable of learning new skills during their careers becomes increasingly pressing [1, 2]. In the Singapore context, where this study will take place, Singapore’s SkillsFuture programme, promoted by the Ministries of Education, Manpower, and Trade & Industry, aptly recognises that societies need workers with the capacity for flexible behaviour. That is, the ability to adapt to change, problem-solve in new situations based on previous experience to achieve in jobs that are likely to emerge over the next few decades [3]. This individual capacity for cognitive flexibility is central to the modern digital age with its rapidly changing settings at home and work [4, 5]. High cognitive flexibility is associated with improved wellbeing which will promote a flourishing society and will lead to improved social and workplace outcomes [6, 7] as well as entrepreneurship and innovation [8].

As we recognise the need for flexible behaviour and transferable skills in our workforce, we need our education systems to equip citizens with the cognitive flexibility they need to develop these skills for the future [9, 10]. However, there currently need to be more evidence-based training programmes that can effectively support and promote cognitive flexibility across the life course.

### Cognitive flexibility

Cognitive Flexibility (CF) is a component of executive function (EF), along with working memory and inhibitory control [11]. Existing studies encourage the view that there is something quite specific about the flexibility construct, making it separable from the other main EF components. The evidence for dissociations of CF and other executive functions comes from psychometric studies, genetic and neuroscientific investigations, and clinical findings (for recent reviews, see [12, 13]).

From the psychometric perspective, there is a general, well-known problem in measuring components of EF, which is that of "task impurity". Most cognitive tasks involve non-executive functions and commonly involve different loadings on its three prime components. Virtually all tasks have a working memory load, and most involve a degree of response inhibition. Importantly, Friedman and Miyake found that working memory updating has good relationships with measures of IQ (relevant to the general factor, *g*), but CF did not correlate highly with IQ measures [14]. Their recent review concluded, "all the studies we reviewed found evidence that shifting was separable from updating or working memory in older children and adults" [11]. They found that when inconsistencies did arise, they tended to involve the inhibition factor. Regarding relationships of shifting with response inhibition, Blackwell et al. found that children who were better at card sorting were worse at response inhibition [15]. A similar dissociation has been found for adults raised in stressful environments [16]. Therefore, although EF has a certain unity, there is also diversity, especially concerning CF.

Given the issue of "task impurity", to effectively measure the psychological construct of cognitive flexibility (as separate from other executive functions), it is necessary to administer multiple CF tests that load on the component of interest and to adopt a latent variable approach to analysis. With this method, Friedman and Miyake showed clear evidence of the separability of CF from other EFs [14]. This study showed that tests of CF ("shifting"), inhibition, and working memory have relatively low correlations with one another (representing an overall EF construct) but correlate well within each component. However, what is unknown–and would be a novel scientific advance–is whether cognitive flexibility itself may be further fractionated into sub-components (such as relating to rule learning and exploration as distinct from executive switching) and the extent to which different CF tasks tap into these sub-skills. In the current project, we seek to address this gap in understanding the nature and measurability of the cognitive flexibility construct to generate robust operational measures of CF for our later training studies.

In contrast to working memory and inhibition control, which are highly heritable skills, cognitive flexibility (CF) may be influenced more by environmental factors and potentially beneficial from training more effectively [11, 17]. Previous CF training interventions have used tasks that activate other executive functions, making them less precise as training tools [17–19]. To develop a training approach more precisely targeted at CF, we adopt the Structure Learning (SL) task, which involves identifying patterns in the presentation of stimuli without explicit feedback [20–23], as a foundational training paradigm (for more details about the SL task, see Methods section and Appendix D in S1 File). The SL training approach emphasizes the emergence of flexibility through exploring and rule learning generated in dynamic environments. SL training might lead to higher-order and potentially generalizable "learning-to-learn" abilities rather than rote memorization of specific information. We hypothesize that SL performance is associated with CF measures; thus, we include SL in the task battery and carefully examine its relationship with CF and other cognitive constructs.

A growing literature demonstrates links between CF and key outcomes of interest, particularly academic performance. For example, Yeniad et al. found that associations between shifting ability and reading and maths performance were "substantial and significant" [24], and Mayes et al. found that performance on the Wisconsin Card Sort Test–a prototypical test of cognitive flexibility was one of the very few measures that predicted maths performance, after accounting for general intelligence [25]. Accumulating data from these and other studies have provided largely correlational evidence and, to a lesser extent, developmental and intervention-based evidence for links between cognitive flexibility and “real-world” outcomes. However, few studies have adequately controlled for the effects of IQ and working memory (or inhibition) or adopted a latent variable approach to elucidate the specific contribution of cognitive flexibility toward the outcomes of interest. Therefore, in this new project, we seek to better characterise the relationship between the CF construct and academic outcomes, creativity, problem-solving and socioemotional skills in the context of Singaporean young adults.

### Research aim and strategy

Prior research has signified CF’s theoretical and practical importance as a vital factor in optimising success. To better characterise the CF construct and clarify CF’s cognitive and social underpinnings, we proposed the current project to investigate the relationships between CF, other executive functions, and other critical cognitive and social constructs in a Singaporean cohort. Specifically, the study aims to confirm whether there is a relative dissociation between CF and other executive functions (such as working memory and inhibition). We further aim to assess relationships between CF and other primary outcome variables, including structure learning, creativity, literacy, numeracy, and problem solving. Finally, the research aims to assess the influence of vital socio-cognitive variables (e.g., multilingualism, perceived social support, tolerance of uncertainty, and social decision-making) on these relationships. Understanding these relationships has value for translational research, such as in organisational settings (e.g., decision-making, career), and informs the need for CF training and development in the local educational curricula. The main hypotheses are summarized below.

1. Latent variable analyses will show relative dissociation between cognitive flexibility and other executive functions (such as working memory and inhibition).
2. Cognitive flexibility will be relatively dissociated from general intelligence.
3. Cognitive flexibility will be best represented by more than one latent variable.
4. Structure learning scores will be associated with at least one of the cognitive flexibility latent variables.
5. Cognitive flexibility latent variable(s) will be associated with at least one primary outcome variable (creativity, literacy, numeracy, and problem solving)
6. The relationship between structure learning scores and cognitive flexibility will vary significantly at different levels of each primary socio-cognitive variable (multilingualism, perceived social support, tolerance of uncertainty, and decision-making factors).

The current study is part of a large-scale international collaboration under the University of Cambridge-Nanyang Technological University Centre for Lifelong Learning and Individualised Cognition (Cambridge-NTU CLIC). CLIC launched as a flagship research programme within the National Research Foundation of Singapore’s Science of Learning initiative and aims to improve support for lifelong learning and cognitive agility. The following protocol describes an adult characterisation study which aims to investigate the construct validity of CF and CF’s relationship with other cognitive constructs of interest. Thereafter, this study will be referred to as the “CLIC adult study”, as distinct from a related study (not reported here) that will be conducted with adolescents. Results of this study will inform the development of intervention paradigms to enhance CF (i.e., through structure learning-based training). CLIC projects also include adolescent studies and intervention studies, which will be reported separately.

## Methods

### Methods overview

**Fig 1:**
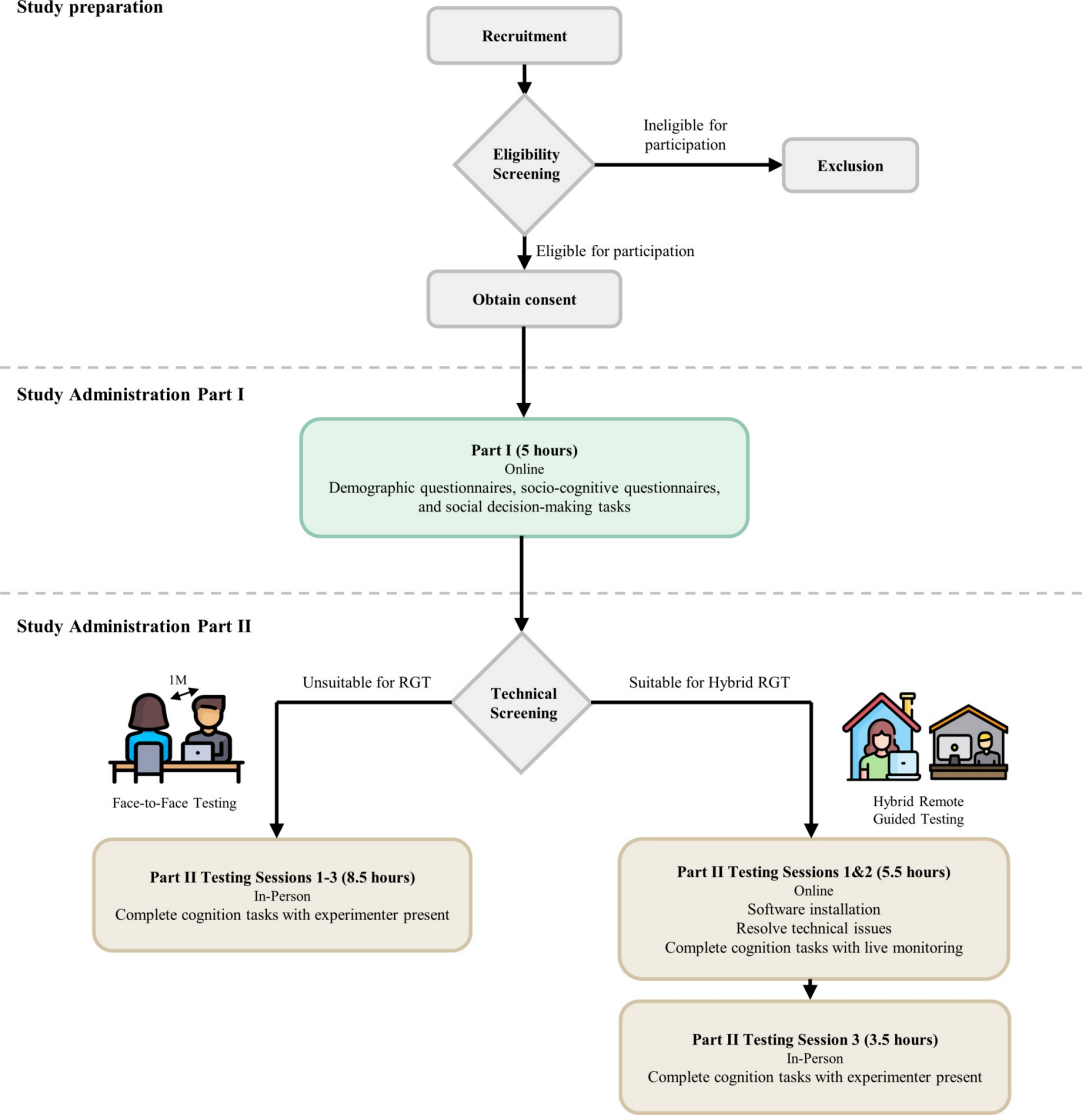
Overall workflow of the CLIC Phase 1 adult study. Detailed documentations of all tasks and questionnaires can be found in Appendices A to F in S1 File.

The CLIC adult study protocol will be described in three main sections: Study Preparation, Study Administration Part I (Demographics, social questionnaires, and social decision-making tasks), and Part II (Cognitive tasks). Fig 1 summarises the overall study workflow. Study preparation encompasses recruitment, eligibility screening, and consent procedures. Study Administration Part I is delivered online, including demographics, socio-cognitive questionnaires, and social decision-making tasks. Part II of the study, the cognitive task battery, is administered in a lab or a hybrid remote-guided testing setting, based on participants’ preferences and suitability for online task administration.

### Study preparation

#### Ethics

This study has obtained ethical approval (IRB-2021-761 "Executive Functions and Learning") from the Nanyang Technological University Institutional Review Board (IRB). Written consent from all participants will be obtained for this study.

#### Recruitment overview

Participants in this study will be Singaporeans recruited from vocational institutions (such as the Institute of Technical Education), institutions of Higher Education (NTU, other local universities and polytechnics), as well as from the general working population via community sampling (e.g., community centres, clinics, via social media platforms). Interested participants will be provided with a study sign-up webpage. The recruitment phase will commence on November 22, 2021, and will continue until January 22, 2024, or until the desired sample size has been reached, whichever occurs first. Access to information that could identify individual participants will only be granted to the Principal Investigator, Co-Principal Investigators, and key research staff assigned to manage personal data, such as for contacting the participant to schedule for an experiment.

#### Eligibility screening and consent

Upon signing up for the study, participants are requested via email to complete the eligibility screening questionnaire (Appendix A in S1 File) online. Participants’ responses to the screening questionnaire will be used to determine their eligibility for this study. See Appendix A in S1 File for the detailed inclusion criteria. Eligible participants will have the study information sheet and consent form sent to them via Adobe sign. They will have their queries or concerns answered through email or a virtual meeting with a research staff before consenting to the study. Afterwards, the participant will be assigned a participant ID for the study.

#### Sample size

We aim to recruit up to 400 healthy young adults aged 18 to 30 years old inclusive, to achieve a final sample size of at least N = 347. This sample size calculation is based on a meta-analysis by Yeniad et al. [24], with a data structure that is similar to the present study, suggesting a tight distribution of pairwise correlations ranging between 0.2 and 0.3 (with isolated extremes 0.09, 0.36). Based on this meta-analysis, we conservatively expect an effect size of approximately r = 0.15. Fisher’s Z transformations would hence require an N of 347 to achieve 80% power with an alpha set at 0.05. Allowing for a 15% data attrition rate, this yields a recruitment target of N = 400.

#### Pre-registration

The current study protocol elaborates on our submitted pre-registration to the Open Science Framework [26].

### Study administration

Table 1 summarises the planned CLIC adult study administration, which comprises of two parts: Part I will be conducted online and Part II will be conducted either entirely in a lab (Face-to-Face, F2F condition) or in a hybrid Remote-Guided Testing setting (Hybrid RGT). All participants will first complete online socio-cognitive questionnaires and social decision-making tasks in Part I (details in section on Social Factors, and in Appendix B in S1 File). The items within each questionnaire/task will be presented in a fixed order, except for one section from the Clip-Q Singapore Language History Questionnaire.

**Table 1:** Study administration Part I and Part II summary.

	Part I	Part II
**Number of lab visits**	0	1 (Hybrid RGT), 2–3 (F2F)
**Test sessions**	Demographics, social questionnaires, and social decision-making tasks	Cognitive task battery
**Constructs of interest tested**	Demographics, multilingualism, perceived social support, tolerance of uncertainty, and social decision-making	Cognitive flexibility, working memory, response inhibition, general intelligence, creativity, literacy, numeracy, problem-solving
**Total time**	5 hours, over 21–27 days depending on participant’s schedule	8.5–9 hours, completed over 3 sessions
**Venue**	Home-based administration (Online)	Laboratory and online

**F2F:** Face-to-Face testing; **RGT**: Remote Guided Testing. Estimated durations include study set up, briefing, and breaks.

Part II can be administered flexibly via one of two testing modalities: Face-to-Face in-lab testing (F2F) and hybrid Remote-Guided Testing (RGT). To tackle the challenges posed by the global COVID-19 pandemic in conducting human psychological research, Leong et al. proposed a new mode of testing–Remote-Guided Testing, as a promising alternative to the traditional in-person testing mode while maintaining the quality of cognitive data collected [27]. With the easing of social distancing measures, the CLIC Cognition Team has adapted the RGT protocol originally developed in 2020 to a hybrid-RGT version in 2022, incorporating both in-person and online testing.

At the beginning of Part II, participants will complete a technical questionnaire to ascertain their eligibility for RGT (See Fig 1. Full questionnaire can be found in Appendix C in S1 File). The screening criteria include operating system compatibility for our testing software, minimum screen size and resolution, and wired keyboard and mouse connections. If the participant meets the necessary technical criteria, arrangements will be made to schedule them for the hybrid-RGT sessions, otherwise, they will be scheduled for F2F sessions.

After confirming the participants’ three sessions in Part II of the study, they will complete the same set of cognitive tasks. The cognitive task battery will be delivered both manually and on computerised study platforms, such as Cambridge Neuropsychological Test Automated Battery (CANTAB) (www.cambridgecognition.com), Inquisit 6 (Millisecond, 2019), Gorilla Experiment Builder (www.gorilla.sc), and iABC (iabc.psychol.cam.ac.uk/welcome), as detailed in the next section and in Appendix D in S1 File. The task orders will be deliberately randomised to control for order effects, depending on the allocation of tasks within each session. Participants will be randomly assigned to one of the three testing orders, as summarised in Appendix E in S1 File. The F2F sessions will be administered in a lab setting using a ThinkPad E14 laptop, wired mouse and earphones. The entire cognitive task battery, tested over three sessions, will take approximately 8.5–9 hours to complete. Appendix F in S1 File summarises the estimated task durations for each cognitive task.

### Comprehensive task battery

**Fig 2:**
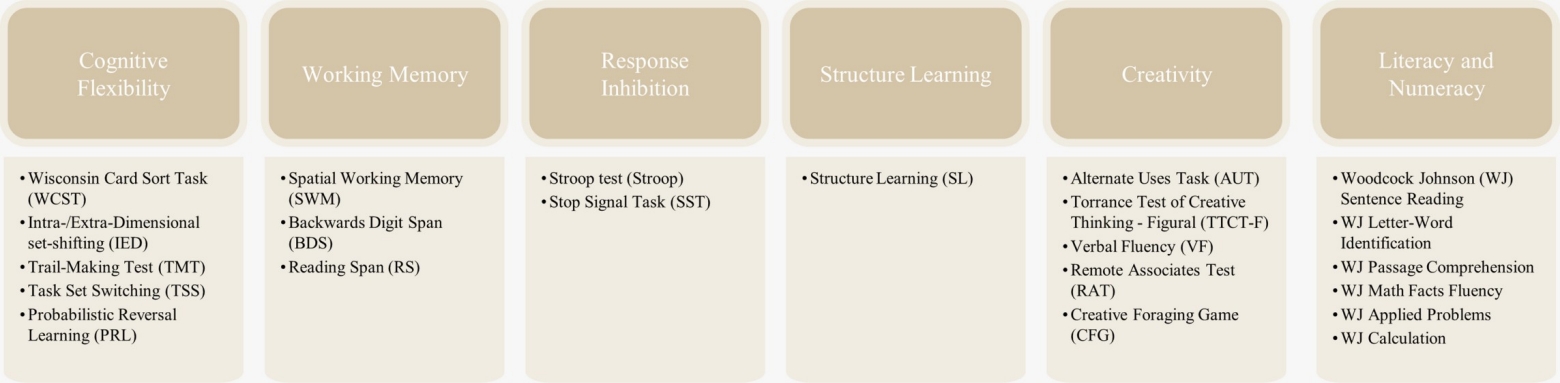
Cognitive tasks administered in the CLIC adult study (Part II), summarised by construct. Appendix D in S1 File documents the full task details.

To address the study’s research hypotheses, we curated a comprehensive test battery consisting of cognitive tasks, social and multilingualism questionnaires, and decision-making tasks. We aim to measure critical cognitive constructs such as cognitive flexibility, working memory, response inhibition, structure learning, general intelligence, creativity, literacy, numeracy, problem-solving, as well as socio-cognitive variables such as multilingualism, social decision-making, perceived social support, tolerance of uncertainty (need for closure and social experiences), and career decision making and planning. Fig 2 summarises the primary cognitive constructs examined within the CLIC adult study, which are further described in the following text and Appendix D in S1 File.

#### Cognitive flexibility

**Fig 3:**
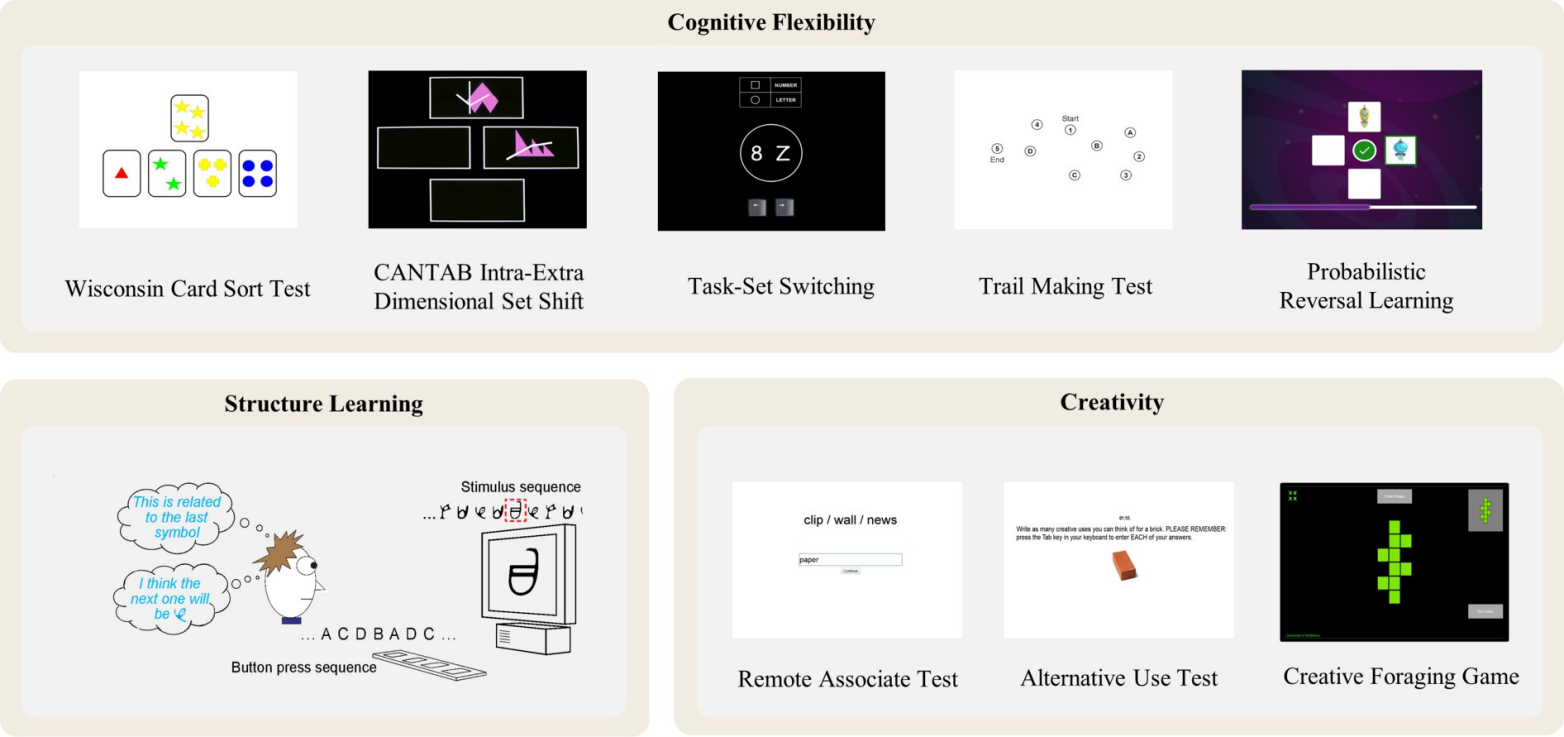
Illustration of a subset of cognitive tasks used in the CLIC adult characterization study. For key constructs of interest, such as cognitive flexibility and creativity, we administer multiple tasks to improve measurement validity. See Appendix D in S1 File for the full task list and details.

One major aim of this study is to validate the construct of cognitive flexibility (CF) as a latent variable, before establishing its relationship to structure learning (SL) and other outcome variables of interest. To characterise the CF construct, we curated five tests (see Fig 3). The Wisconsin Card Sort Test [28] and CANTAB Intra-/Extra-Dimensional Set Shifting task [29] probe set-shifting abilities, in which participants need to learn the rule via feedback and overcome the old rule when there is a rule change. The Trail Making Test [30] and Task Set-Switching [31] paradigm are classic assessments of executive switching abilities, in which participants need to follow the instructed rule to switch between different actions. Similar tests have been used previously to assess the CF construct [13, 32]. A fifth test, Probabilistic Reversal Learning [33], has excellent face validity as a test of CF, but requires decision-making under uncertainty to a greater extent than the other four tests, because the task rules are probabilistic rather than deterministic. This uncertainty might offer more room for exploration and thus tap into a unique aspect of CF that could be related to implicit learning of probabilistic patterns and creativity.

#### Structure learning

The Structure Learning (SL) task [20, 21] involves identifying patterns in seemingly stochastically presented symbols and making predictions based on learned patterns. On each trial, participants will see a sequence of visual symbols and will then be asked to predict which symbol they think should come next in the sequence (Fig 3, middle left panel). The symbol sequences are embedded with pre-defined probabilistic contingencies. Studies have shown that participants’ responses will gradually reflect those hidden probabilistic patterns through SL training, even without trial-level feedback [20].

The SL task tests a person’s ability to implicitly learn and understand the underlying structure of their environment under conditions of uncertainty and adjust to new problem-solving rules based on prior experience and current information. The current study hypothesizes that the learning strategies used in the SL task may be related to CF and aims to examine the relationship between CF and SL task performance to inform the development of future CF training interventions.

#### Outcome variables

The key outcomes of interest in relation to CF include creativity, mathematics, and language skills. To assess creativity (Fig 3), we will employ classical tests of convergent and divergent creativity, including the Remote Associates Test, Alternative Uses Test, Torrance Test of Creative Thinking—Figural, as well as basic verbal fluency (phonological and semantic) measures. We will also include a novel test, the Creative Foraging Game to measure explore-exploit decisions in creative foraging behaviours [34]. We will use the Woodcock Johnson IV: Tests of Achievements [35] reading (Letter-Word Identification, Sentence Reading Fluency, Passage Comprehension) and maths (Calculation, Applied Problems, Maths Facts Fluency) subtests as standardised measures of language and maths skills.

We will also examine how these cognitive factors relate to real-life, impactful, and significant decisions and phenomena. Given the increased interest in an adaptive workforce (see introduction) we focus here on career decision making in university students, as this population will soon be facing an important career and life transition to employment. We specifically examine the general phenomenon of career construction and career flexibility–i.e., the way individuals construct, adapt and change their vocational and career identity to respond to a dynamic work environment. We adopt the Career Construction Model of adaptation [36, 37].

#### Social factors

**Fig 4:**
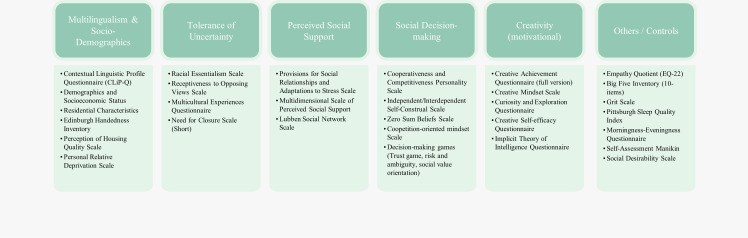
Main socio-cognitive questionnaires and decision-making tasks administered in the CLIC Phase 1 study (Part I), summarized by construct. See Appendix B in S1 File for the full list.

Individuals do not learn in a vacuum–the socio-cultural environment and dynamics might enhance, promote, or inhibit the development and manifestation of executive functions. This is true for more systemic environmental factors such as socio-economic status and formal education but has even been found for engagement in particular activities such as music making [38–40]. Indeed, social interactions with peers, elementary school social experiences, social skills intervention programs and even a very short social exchange have been associated with executive functions and their development [41–43]. We therefore need to characterise participants’ social context in order to understand how these might moderate the cognitive factors of interest. For the current study, we focus on four key factors for which the literature provides strong indications of a possible link with cognitive flexibility and that are relevant in the local context (see Fig 4, which also includes complementary questionnaire measures of creativity and control variables).

***Multilingualism*** is the ability of an individual speaker or a group of speakers to communicate effectively in more than one language. There is overwhelming evidence that the languages in a multilingual’s repertoire are always jointly activated even if not required in a given context. This results in constant competition and depending on the interlocutor there will be a need for the selection of the target, and inhibition of the non-target language (in monolingual contexts) or switch frequently between languages (multilinguals). These skills were originally mostly thought to link with inhibition but are more recently seen as a broader issue of executive attention, which includes most of the executive functions [44]. The Singapore government actively promotes multilingualism resulting in most people in the country being able to speak or at least understand multiple languages and the environment also presenting multilingually.

***Tolerance of uncertainty*** refers to the extent to which someone is motivated (as opposed to being able) to engage in unconventional and novel ways of thinking and doing. We focus on social aspects here as social contexts are typically full of ambiguity, involving opposing views and unpredictable others producing feelings of uncertainty. Specifically, we examine the need for cognitive closure [45], the willingness to accept opposing views [46], beliefs about the fundamental and unchanging nature of race (racial essentialism) [47], and the extent of multicultural experiences someone has [48]. Thus, we supplement the examination of the capacity for cognitive flexibility with the motivational desire for a definite and crystallised answer, as opposed to ambiguity (need for closure), and the tendency to apply similar motivations to cognitions about social groups (racial essentialism). We also will capture people’s experience with social situations that may generate uncertainty and challenge conventions (e.g., multiculturalism, willingness to accept opposing views).

***Perceived social support*** indicates to what extent an individual believes that they can rely on their social contacts especially during times of need. Social exchanges are built on the expectation of reciprocity–it is thought that confidence in the levels of social support may promote openness and flexibility. Singaporeans have multiple sources of social support, including an *extended* family, friends and others in a tight-knit community, possible larger and more diverse than in Western societies. We measure perceived social support based on individuals’ perceptions of the extent to which they have significant others, family and friends they can rely on in terms of need [49, 50] as well as their social networks [51].

***Social Decision-Making*** preferences reflect the way participants choose between options necessitating to take into account the preferences and strategies of other people. Multidisciplinary research early has suggested that human intellect and executive functions, to a great extent, evolve as a response to social exchanges, and especially balancing between cooperation and competition [52–54]. Such dynamic and intense social interactions require flexible thinking and adaptive responses to allow for mentalising, perspective taking, learning of norms and exercising strategic thinking and behaviour These preferences allow for the building of trust and reciprocity, the pillars of efficient economic exchanges [55, 56] and entrepreneurial cognition [57]. These factors are also examined here with stylized game-theoretic approaches.

The questionnaires included some additional measurements. The expression of creativity depends not only on the creative potential (measured by the creativity tasks in Part II–see Fig 2) but, as well, the beliefs and motivation to be creative (“creative mindset”). We thus added questionnaires that measure these beliefs [58–62]. Finally, a set of control variables was added, including personality (BIG-5 and Empathy Quotient) as well as measurements of sleep quality and preferences (Fig 4).

## Analysis plan

Our large-scale data collection and comprehensive task battery permits the use of SEM methods to address the aforementioned "task impurity" issue [32]. Accordingly, we plan to establish measurement models of latent constructs (e.g., CF, other EFs, creativity, and social factors) and test the hypothesized relationships between the latent constructs. Further, computational modelling can reveal potentially separable cognitive processes with formal parameterised models [63], offering an in-depth look at the cognitive constructs of interest. Computationally modelled parameters from the Reinforcement Learning (RL) framework utilise trial-level data and their relationships with task feedback to separate participants’ characteristics in value updating and decision-making. RL modelling parameters thus provide insights into mechanistic links between CF and other cognitive constructs that may otherwise be hidden when investigated using surface-level behavioural indices (e.g., overall accuracy, mean response time). In the current study, tasks that involve rule-learning via feedback can be modelled using the RL framework [64–66]. In particular, we plan to use hierarchical Bayesian RL models to extract learning and explore-exploit related parameters from Wisconsin Card Sort Task [67], CANTAB Intra-/Extra-Dimensional Set Shifting task [68], and Probabilistic Reversal Learning task [69]. We also plan to apply task-specific computational models where appropriate, e.g., structure learning task [20, 21] and stop signal task [70].

## Significance and impact

The current study will be the first systematic large-scale investigation into the construct validity of CF and its interrelationship with other important cognitive skills such as learning and creativity, in an Asian context. A further point of novelty is that the study will offer an in-depth analysis of CF sub-components and their specific relation to other cognitive skills, in particular structure learning and creativity. The exploration of CF as a non-unitary construct will be a novel theoretical contribution to the field of executive functions. Finally, the results of the study are expected to be useful for informing classroom pedagogy and the design of lifelong learning policies and curricula, suited for the Singaporean context but also with wider applicability. Here, we will adopt the Remote-Guided Testing (RGT) approach to overcome challenges posed by any on-going COVID-19 pandemic-related restrictions. Leong et al. demonstrated equivalent data quality from the RGT method compared with lab-based settings [27]. Thereby, there is potential for the RGT method to complement traditional F2F methods in both research and clinical settings, particularly in situations where in-person meetings would be difficult or impossible. In clinical settings, remote testing methods not requiring the use of personal protective equipment such as masks may be beneficial to reduce the communication barrier between experimenter and participant [71].

CLIC is unique in its focus on translational research—bringing cognitive neuroscience knowledge into the design of learning pedagogies and lifelong learning programmes. Cognitive flexibility may be key to learning and the ability to upskill, reskill, and fit existing knowledge to ever-evolving life situations, such as job changes and environmental uncertainties [72, 73].

The current study also has a unique potential impact on education in both school and life-long learning settings. Globalisation and rapid technological development have provided myriad opportunities for the advancement of student education and novel training in the workplace. However, these same processes will also engender new sources of uncertainty, requiring students to develop a fluid mindset to respond to accompanying challenges. Critical competencies such as creativity, curiosity, and grit are some of the traits that can help students navigate this uncertainty and embrace the challenges [74, 75]. For example, students today are already constantly bombarded with many sources of information on the Internet, and they need to develop capacities to analyse these data systematically. Without the ability to fluidly transition between similar but competing ideas, students would effectively be perseverating on familiar interpretations of these data. Navigating through a high-density information environment requires intelligence and, more importantly, the ability to discover new patterns amid the noise and flexibly disengage with previously valued methods. Thus, cognitive flexibility is the critical skill that may be important to nurture among students and life-long learners.

## Limitations and future directions

The current testing protocol is adapted for adults with testing modalities in-lab or in a standardised remote guided testing scenario. While this helps with collecting high-quality data, there are limitations when generalizing to other populations or testing scenarios. For example, our protocol needs age-appropriate adaptation when testing younger age groups (e.g., infants and adolescence), which require additional considerations in terms of testing duration, language requirement, and cognitive load; or older age groups, such as busy working professionals (that might not have the time or motivation for face to face testing) or seniors (over 65 years old with particular socio-cognitive or even physical / movement limitations).

Finally, there is potential to enhance participant motivation and engagement with the cognitive tasks through gamification, including social gamification (such as cooperative and competitive incentive schemes). Adding game elements could enhance participants’ motivation and engagement for a wide age range [76–78]. Applying gamification elements judiciously could lead to the creation of enjoyable and scientifically accurate cognitive evaluations. Identifying suitable elements for gamification is a secondary aim of the project, which will increase the potential translational value and impact of our findings.

## Supporting information

S1 File Appendices A to G for “Study protocol: How does cognitive flexibility relate to other executive functions and learning in healthy young adults?”. (DOCX)
