# Supplementary material for: Study protocol: How does cognitive flexibility relate to other executive functions and learning in healthy young adults?
Source: PLoS One. 2023 Jul 20;18(7):e0286208. doi: 10.1371/journal.pone.0286208 (PMC10358919; doi:10.1371/journal.pone.0286208)
Supplement: S1 File — (DOCX) [file pone.0286208.s001.docx]

# Appendices A to G for “Study Protocol: How Does Cognitive Flexibility Relate to Other Executive Functions and Learning in Healthy Young Adults?”

## S1 Appendix A. Eligibility screening questionnaire and inclusion criteria

| 1. Please indicate your ID in the textbox below. |  |
| --- | --- |
| 1. Are you a Singaporean? | Inclusion criteria: ‘Yes’ |
| 1. What is your date of birth? (dd-mm-yyyy). | Inclusion criteria: individuals aged between 18-30 |
| 1. Do you have basic English comprehension skills (i.e., able to read, understand, and/or write in basic English)? | Inclusion criteria: ‘Yes’ |
| 1. What is your sex?    1. Female    2. Male | Inclusion criteria: any option chosen |
| 1. What is your race/ethnicity? 2. Chinese 3. Malay    1. Indian    2. Others, please specify: ___________ | Inclusion criteria: any option chosen, but might become a screening criterion if diversity in the participants’ profiles has not been reached during the course of the study |
| 1. Please indicate your mother tongue.    1. Chinese    2. Malay    3. Tamil    4. Others, please specify: ____________ | Inclusion criteria: any option chosen, but might become a screening criterion if diversity in the language profiles of participants has not been met over the course of the study |
| 1. Are you currently a full-time student? Please select the option that most accurately describes you. 2. Yes, I am a full-time university undergraduate student 3. Yes, I am a full-time polytechnic student 4. Yes, I am a full-time ITE student 5. Yes, I am a full-time student at (please specify, if not described above): 6. No, I am not a full-time student | [Inclusion criteria: ‘Yes’ for student sample]  [If ‘Yes, I am a full-time university undergraduate student’ is selected, proceed to Questions 911 then 19;  If ‘Yes, I am a full-time polytechnic student’ is selected, please proceed to Questions 12-14 then 19;  If ‘ Yes, I am a full-time ITE student’ is selected, please proceed to Questions 15-17 then 19;  If ‘No, I am not a full-time student’ is selected, skip to Question 18]1; If all other options are selected, skip to Question 12] |
| 1. If you are a full-time university undergraduate student, which year are you presently in? 2. Year 1 3. Year 2 4. Year 3 5. Year 4    1. Year 5 or above    2. Others, please specify: | Inclusion criteria: any option chosen, but might become a screening criterion if there is an uneven spread of participants based on year of study during the course of the study |
| 1. How long (in years) is your university program? __________ | Inclusion criteria: any response provided |
| 1. If you are a university student, please indicate the school/ discipline you are from.   **NTU**   1. Nanyang Business School [Non-STEM] 2. College of Engineering [STEM] 3. College of Humanities, Arts and Social Sciences [Non-STEM] 4. College of Science [STEM] 5. Lee Kong Chian School of Medicine [STEM] 6. Sport Science & Management [Mixed] 7. National Institute of Education (Arts Education) [Non-STEM] 8. National Institute of Education (Science Education) [STEM] 9. Others, please specify:   **NUS**   1. Faculty of Law [Non-STEM] 2. School of Medicine [STEM] 3. Faculty of Dentistry [STEM] 4. School of Design & Environment [Mixed] 5. Faculty of Engineering [STEM] 6. School of Computing [STEM] 7. Faculty of Engineering & School of Computing [STEM] 8. Faculty of Science [STEM] 9. Business School [Non-STEM] 10. Faculty of Arts & Social Sciences [Non-STEM] 11. Faculty of Arts & Social Sciences & Faculty of Science [Mixed] 12. Yong Siew Toh Conservatory of Music [Non-STEM] 13. Others, please specify:   **SMU**   1. School of Accountancy [Non-STEM] 2. Lee Kong Chian School of Business [Non-STEM] 3. School of Economics [Non-STEM] 4. School of Computing and Information Systems [STEM] 5. Yong Pung How School of Law [Non-STEM] 6. School of Social Sciences [Non-STEM] 7. Others, please specify:   **SIT**   1. Engineering [STEM] 2. Chemical Engineering & Food Technology [STEM] 3. Infocomm Technology [STEM] 4. Health and Social Sciences [Mixed] 5. Design and Specialised Business [Non-STEM] 6. Others, please specify:   **SUSS**   1. S R Nathan School of Human Development [Non-STEM] 2. School of Business [Non-STEM] 3. School of Humanities and Behavioural Sciences [Non-STEM] 4. School of Law [Non-STEM] 5. School of Science and Technology [STEM] 6. Others, please specify:   **SUTD**   1. Architecture and Sustainable Design [STEM] 2. Design and Artificial Intelligence [STEM] 3. Engineering Product Development [STEM] 4. Engineering Systems and Design [STEM] 5. Information Systems Technology and Design [STEM] 6. Science, Mathematics and Technology [STEM] 7. Humanities, Arts and Social Sciences [Non-STEM] 8. Others, please specify: | Inclusion criteria: any option chosen, but might become a screening criterion if there is an unequal number of STEM vs non-STEM participants over the course of the study |
| 1. If you are a full-time polytechnic student, which year are you presently in? 2. Year 1 3. Year 2 4. Year 3 5. Year 4 6. Others, please specify: |  |
| 1. How long (in years) is your polytechnic program? __________ | Inclusion criteria: any response provided |
| 1. If you are a polytechnic student, please indicate the school/ discipline you are from.   **SP**   1. School of Architecture & the Built Environment [Mixed] 2. School of Business [Non-STEM] 3. School of Chemical & Life Sciences [STEM] 4. School of Computing [STEM] 5. School of Electrical & Electronic Engineering [STEM] 6. School of Life Skills & Communication [Non-STEM] 7. Media, Arts & Design School [Non-STEM] 8. School of Mechanical & Aeronautical Engineering [STEM] 9. Singapore Maritime Academy [Mixed] 10. Others, please specify:________   **NP**   1. Applied Sciences [STEM] 2. Built Environment [Mixed] 3. Business & Management [Non-STEM] 4. Engineering [STEM] 5. Health Sciences [Non-STEM] 6. Humanities [Non-STEM] 7. Information & Digital Technologies [STEM] 8. Media & Design [Non-STEM] 9. Maritime Studies [Mixed] 10. Others, please specify:________   **NYP**   1. School of Applied Science [STEM] 2. School of Business Management [Non-STEM] 3. School of Design & Media [Non-STEM] 4. School of Engineering [STEM] 5. School of Health & Social Sciences [Non-STEM] 6. School of Information Technology [STEM] 7. Others, please specify:________   **TP**   1. School of Applied Science [STEM] 2. School of Business [Non-STEM] 3. School of Design [Non-STEM] 4. School of Engineering [STEM] 5. School of Humanities & Social Sciences [Non-STEM] 6. School of Informatics & IT [STEM] 7. Others, please specify:________   **RP**   1. School of Applied Science [STEM] 2. School of Engineering [STEM] 3. School of Hospitality [Non-STEM] 4. School of Infocomm [STEM] 5. School of Management and Communication [Non-STEM] 6. School of Sports, Health and Leisure [Non-STEM] 7. School of Technology for the Arts [Non-STEM] 8. Others, please specify:________ | Inclusion criteria: any option chosen, but might become a screening criterion if there is an unequal number of STEM vs non-STEM participants over the course of the study |
| 1. If you are a full-time ITE student, which year are you presently in?    1. Year 1    2. Year 2    3. Year 3    4. Year 4    5. Others, please specify: |  |
| 1. How long (in years) is your ITE program? __________ | Inclusion criteria: any response provided |
| 1. If you are an ITE student, please indicate the school/ discipline you are from.   **ITE College Central**   1. School of Business & Services [Non-STEM] 2. School of Electronics & Info-Comm Technology [STEM] 3. School of Engineering [STEM] 4. School of Design & Media [Non-STEM] 5. Others, please specify:________   **ITE College East**   1. School of Business & Services [Non-STEM] 2. School of Electronics & Info-Comm Technology [STEM] 3. School of Engineering [STEM] 4. School of Applied & Health Sciences [STEM] 5. Others, please specify:________   **ITE College West**   1. School of Business & Services [Non-STEM] 2. School of Electronics & Info-Comm Technology [STEM] 3. School of Engineering [STEM] 4. School of Hospitality [Non-STEM] 5. Others, please specify:________ | Inclusion criteria: any option chosen, but might become a screening criterion if there is an unequal number of STEM vs non-STEM participants over the course of the study |
| 1. If you are not a full-time student, are you currently working? Please select the option that most accurately describes you.    1. Working (full-time paid employee)    2. Working (part-time paid employee)    3. Working (self-employed)    4. Not working    5. Other (please specify): | Inclusion criteria: ‘Yes’ for working adults/professionals sample |
| 1. Please indicate if you have ever been diagnosed with and/or are taking medication for any of the following conditions:    1. Developmental/ Learning disorders (e.g., autism spectrum disorder, attention-deficit/hyperactivity disorder, cerebral palsy, dyslexia, enrolment in special education classes etc.)    2. Neurological disorders (e.g., epilepsy, dementia, traumatic brain injury, stroke, insomnia, etc.)    3. Psychiatric disorders (e.g., depression, bipolar disorder, schizophrenia, alcohol or substance abuse, etc.)    4. Color blindness    5. Hearing/ Ear disorders (e.g., hearing loss, tinnitus, ear infection, perforated eardrum, etc.)    6. Any other neurological or psychological disorders and/or taking any psychiatric or neurological medications, if so, please specify: ___________ | Inclusion criteria: ‘No’ for all the above items |
| 1. Have you ever had a head injury or been knocked unconscious? ___________ | Inclusion criteria: ‘No’ |
| 1. Have you ever sustained a concussion? ___________ | Inclusion criteria: ‘No’ |
| 1. Do you have a history of seizures? ___________ | Inclusion criteria: ‘No’ |
| 1. Is your vision normal or corrected-to-normal, such as wearing contact lenses and/or glasses? ___________ | Inclusion criteria: ‘Yes’ |
| 1. How many alcoholic drinks do you generally consume within a month? ___________ | Inclusion criteria: Amount of drinks less than:   1. For men, consuming more than 70 drinks per month 2. For women, consuming more than 35 drinks per month |
| 1. How many cigarettes do you smoke in a day and how long (in years) have you been smoking? ___________ | Inclusion criteria: Less than 20 cigarettes per day |
| 1. Are you currently taking any medications/supplements regularly? If yes, please specify: | Note: Check if participants declare any medication that may have an effect on cognitive function. If have, we will exclude those participants too. |
| 1. Have you ever been diagnosed with a major illness that may have caused brain injury (e.g., stroke, heart attack)? ___________ | Inclusion criteria: ‘No’ |
| 1. Have you undergone any surgery within the past 3 months? ___________ | Inclusion criteria: ‘No’ |
| 1. Do you own a laptop or desktop that you can use for the study? ___________    1. What is the brand of your laptop or desktop that you will be using for the study?       1. Apple, HP, Lenovo, Dell, Acer, Asus, Fujitsu, Others – Please specify: ___________    2. What is the Operating System that your laptop or desktop is on? ___________    3. What is your laptop’s RAM?       1. 4GB, 8GB, 16GB, 32GB, Others – Please specify: ___________    4. Do you have easy and uninterrupted access to the Internet for this laptop or desktop device? ___________ | Inclusion criteria: ‘Yes’  b. Inclusion criteria: Windows 7 or 10, Mac OS  c. Inclusion criteria: 8GB and above  d. Inclusion criteria: ‘Yes’ |
| 1. Which type of voucher would you prefer to receive as compensation for your participation and completion of this study?    1. Capitaland e-voucher    2. Fairprice e-voucher |  |
| 1. For potential bonus payment, which mode of payment do you prefer?    1. Paynow    2. Bank transfer    3. Cash |  |
| 1. Have you received the COVID-19 vaccination?    1. Yes, I have completed 3 doses    2. No, I am not vaccinated    3. Others: ____________ | Inclusion criteria: Yes, I have completed 3 doses |
| 1. Please declare if you have participated in previous CLIC studies in the last 6 months | Inclusion criteria: ‘No’ |

#

## S2 Appendix B. Details of Socio-cognitive questionnaires and social decision-making tasks

### Socio-Cognitive Questionnaires

#### Self-Assessment Manikin

The Self-Assessment Manikin (Bartosova et al., 2019) is a non-verbal pictorial assessment that measures an individual’s emotional response. The original scale consists of three items (representing the valence, arousal, and dominance dimensions) rated on a nine-point scale. Figure B1 shows an example item.


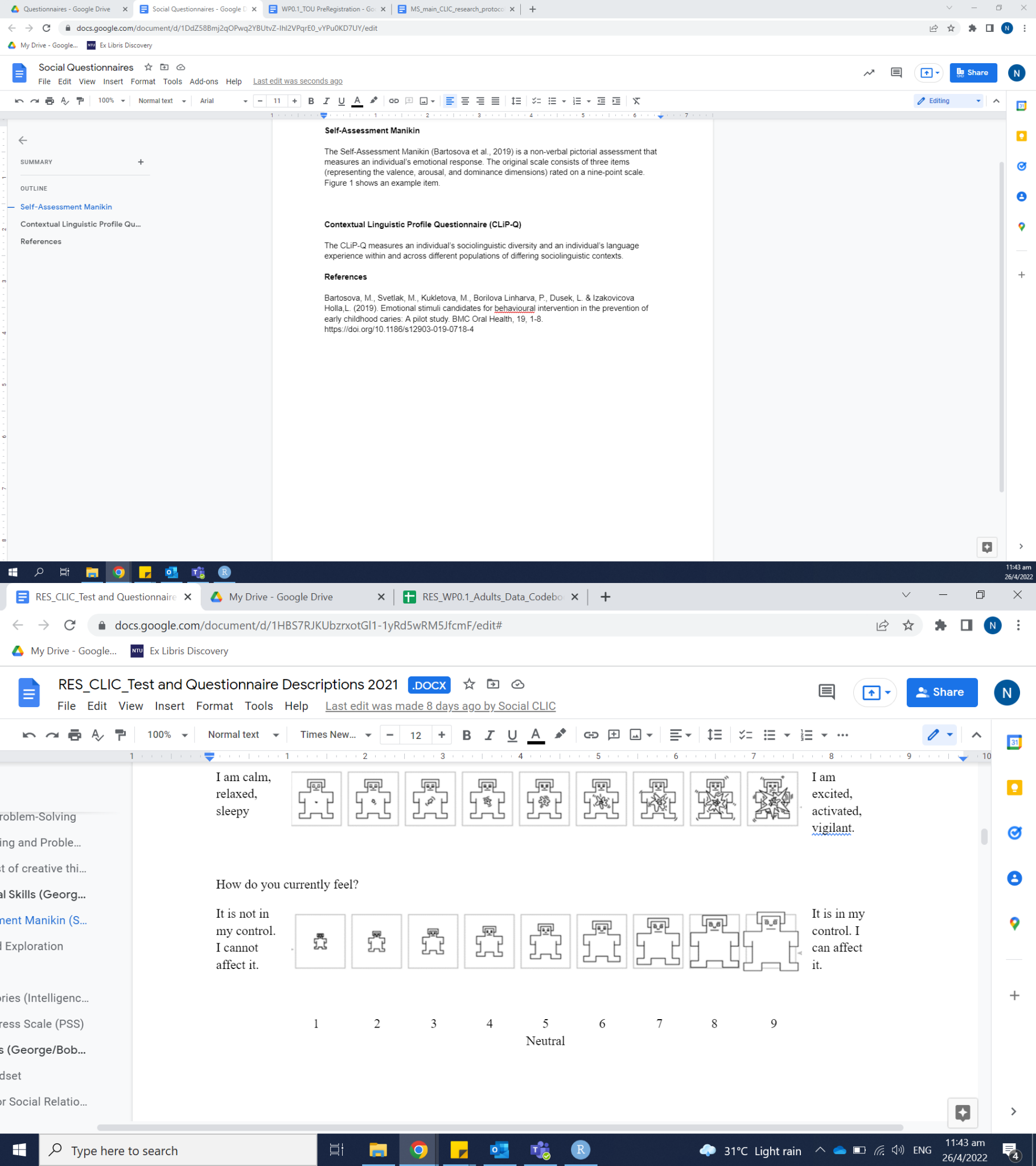


Figure B1: Sample item of Self-Assessment Manikin (dominance item)

#### Contextual Linguistic Profile Questionnaire (CLiP-Q)

The CLiP-Q measures an individual’s sociolinguistic diversity and an individual’s language experience within and across different populations of differing sociolinguistic contexts (Wigdorowitz et al., 2020). It consists of 4 sections measuring basic demographic information, contextual and individual linguistic diversity (including multilingualism in context, in practice and linguistic diversity promotion), language history, use and proficiency, and socioeconomic status. All questions in the questionnaire were carefully assessed and adapted for the Singapore context. For example, for the Singapore version of the questionnaire, multilingualism was measured with items such as “Most people from Singapore can communicate in more than one language,” rated on a scale of 1 = Strongly disagree to 5 = Strongly agree. Proficiency and use were measured for the first three languages that respondents listed in order of acquisition or dominance (in Singapore there were mostly English, Singlish and Mandarin Chinese)(sample items: “At what age did you start using English at school?” “Please select your level of ability from 1 (very low) to 10 (perfect) in the following - Speaking in Singlish”).

#### Edinburgh Handedness Inventory

This is a 12-item self-report questionnaire designed to measure handedness of an individual (Oldfield, 1971). Individuals indicate on a scale of 1 = Left only to 5 = Right only which hand they prefer to use when doing the mentioned activities (sample items: Writing, Drawing, Throwing, Brush teeth).

#### Creative Achievement Questionnaire

This self-report questionnaire measures the respondents’ self-identified prior achievements across 10 creative domains, including visual arts, music, dance, sciences, inventions, etc. (Carson et al., 2005). For each creative domain, the questionnaire presents increasing levels of creative achievement, with the respondent indicating the level of achievement that best represents him/herself on that domain. Sample items include: “I have no training or recognised talent in this area” (all domains), “I play one or more musical instruments proficiently” (music domain), “My work has been cited by other scientists in national publications” (scientific discovery domain).

#### Creative Mindset

This 10-item scale measures respondents’ perceptions of the nature of creativity and consists of two subscales measuring growth mindset and fixed mindset (Karwowski, 2014). Items are measured on a 5-point Likert scale (1 = Definitely not, 5 = Definitely yes). Sample items include: “Everyone can create something great at some point if he or she is given appropriate conditions” (growth mindset), “A truly creative talent is innate and constant throughout one’s entire life”” (fixed mindset).

#### Provisions for Social Relationships and Adaptations to Stress

This measure identifies the degree of social support an individual has in the form of social relationships and consists of 6 dimensions including reliable alliance, attachment, guidance, nurturance, social integration, and reassurance of worth (Cutrona & Russell, 1987). Items are measured on a 4-point scale (1 = Strongly disagree, 4 = Strongly agree). Sample items include: “There are people I can depend on to help me if I really need it” (reliable alliance), “There is no one who shares my interests and concerns” (social integration; reverse-scored).

#### Multidimensional Scale of Perceived Social Support

The Multidimensional Scale of Perceived Social Support (MSPSS) is a brief research tool designed to measure perceptions of support from 3 sources: family, friends, and a significant other (Zimet et al., 1988). The scale consists of 12 items, with 4 items for each subscale, rated on a scale of 1 = Very strongly disagree to 7 = Very strongly agree. Sample items include: “There is a special person who is around when I am in need” (significant other), “My friends really try to help me” (friends).

#### Lubben Social Network Scale

The Lubben Social Network Scale is a 6-item scale designed to gauge social isolation in older adults by measuring perceived social support received by family and friends (Lubben et al, 2006). Sample items include: “How many relatives do you see or hear from at least once a month?” (family), “How many friends do you feel close to such that you could call on them for help?” (friends).

#### Racial Essentialism Scale

This 8-item questionnaire examines an individual’s view and/or understanding of race (No et al., 2008). The items are rated on a 6-point scale (1 = Strongly disagree, 6 = Strongly agree). The questionnaire measures two views on race - the essentialist view of race and the social constructionist view of race. Sample items include: “Races are just arbitrary categories and can be changed if necessary” (social constructionist view), “A person’s race is something very basic about them and it can’t be changed much” (essentialist view).

#### Receptiveness to Opposing Views

Receptiveness to Opposing Views Scale is a 18-item questionnaire that assesses individuals’ receptiveness to opposing views (Minson et al., 2020). It consists of 4 factors (negative emotions, intellectual curiosity, derogation of opponents, sacred issues). Items are rated on a scale of 1 (Strongly disagree) to 7 (Strongly agree). Sample items include: “I find listening to opposing views informative” (intellectual curiosity), “I consider my views on some issues to be sacred” (sacred issues; reverse-scored).

#### Multicultural Experiences Questionnaire

The MEQ is a 15-item self-report questionnaire that measures multicultural experiences and attitudes (Narvaez & Hill, 2010). It assesses the respondents’ level of experience with intercultural contact, such as foreign travel, friendships with cultural/racial outgroup members, knowledge of foreign cultures and mindfulness of discrimination/prejudice. Sample items include: “I travel out of the country (1 = Never, 4 = Regularly),” “I respect the traditions of a culture (1 = Strongly disagree, 5 = Strongly agree).”

#### Brief Pittsburgh Sleep Quality Index

The BPQSI is a 6-item measure examining an individual’s sleep quality and disturbances in the past month (Sancho-Domingo et al., 2021). Sample items include: “During the past month, when have you usually gone to bed at night?” “During the past month, when have you usually gotten up in the morning?” “During the past month, how would you rate your sleep quality overall? (1 = Very good, 4 = Very bad).”

#### Morningness-Eveningness Questionnaire

This is a 5-item self-report questionnaire examining an individual's chronotype (Adan & Almirall, 1991). Sample items include: “During the first half hour after having woken in the morning, how tired do you feel? (1 = Very tired, 4 - Very refreshed)” “At what time in the evening do you feel tired and as a result in need of sleep?”

#### Perception of Housing Quality Scale

This is a 14-item measure of perceived housing quality and measures individuals’ perceptions of how much the physical aspects of their home environment answer to their daily needs and improve their quality of life (Caffaro et al., 2016). Items are either scored for functionality on a scale of 1 (Not at all functional) to 7 (Extremely functional) or the individual’s satisfaction on a scale of 1 (Not at all satisfied) to 7 (Extremely satisfied). Sample items include: “Think about natural lighting in your home and rate it, with regard to your daily needs,” “Think about outdoor air quality and rate it, with regard to your daily needs.”

#### Need for Cognitive Closure Scale

This 15-item short version of the Need for Cognitive Closure Scale (NFCC) is a self-report measure of the respondents’ desire to reach a firm answer, any answer, rather than experience prolonged uncertainty and ambiguity (Roets & Van Hiel, 2011). It consists of 5 dimensions (predictability, close-mindedness, ambiguity, order, & decisiveness). Responses are made on a scale of 1 (Strongly disagree) to 6 (Strongly agree). Sample items include: “I don’t like situations that are uncertain” (ambiguity), “When I have made a decision, I feel relieved” (decisiveness), “I dislike unpredictable situations” (predictability).

#### Personal Relative Deprivation Scale

This is a 5-item questionnaire that measures individual differences in people’s tendencies to feel resentful about what they have compared to what other people like them have (Callan et al., 2011). Items are scored on a 6-point scale (1 = Strongly disagree, 6 = Strongly agree). Sample items include: “I feel resentful when I see how prosperous other people like me seem to be,” “I feel privileged compared to other people like me” (reverse-scored).

#### Perceived Stress Scale

This scale measures an individual's perception of stress, their thoughts and feelings during the last month (Cohen et al., 1994). It contains 10 items rated on a five-point scale (0 = Very often, 4 = Never). Sample items include: “In the last month, how often have you felt that things were going your way?” “In the last month, how often have you felt that you were on top of things?” “In the last month, how often have you been upset because of something that happened unexpectedly?” (reverse-scored).

#### Cooperativeness and Competitive Personality Scale

This is a 23-item questionnaire measuring individuals’ beliefs, behavioural tendencies, and feelings about cooperation and competition (Lu et al., 2013). It consists of six subscales: beliefs about cooperation (cognition), behavioural tendencies about cooperation (behaviour), feelings for cooperation (affect), behavioural tendencies about competition (behaviour), beliefs about competition (cognition), and feelings about competition (affect). Items are rated on a 5-point scale (1 = Do not agree at all; 7 = Totally agree). Sample items include: “I like challenges that are brought by competing with other team members” (competition cognition), “I can usually consider multiple views when I handle tasks” (cooperation behaviour).

#### Independent/Interdependent Self-Construal Scale

This is a 20-item revised version of Singelis’ 24-item Self-Construal Scale (Singelis, 1994) that measures the extent to which the self is construed as independent and/or interdependent from the surrounding social relationships/contexts (Park & Kitayama, 2014). It has two subscales: independent self-construal and interdependent self-construal. All items are rated on a 7-point Likert scale (1 = Strongly disagree; 7 = Strongly agree). Sample items include: “In general I make my own decisions” (independent self-construal), “I am concerned about what people think of me” (interdependent self-construal).

#### Belief in Zero Sum Games Scale

This is a 12-tems questionnaire reflecting beliefs about antagonistic competition over scarce resources (Różycka-Tran et al., 2015). All items are rated on a 7-point Likert scale (1 = Strongly disagree; 7 = Strongly agree). Sample items include: “When someone does much for others, he or she loses,” “Those who give much to others receive much from them” (reverse-scored).

Coopetition-Oriented Mindset Scale This is a sub-scale on coopetition-oriented mindset from the multi-dimensional measure of coopetition (COOP scale) that examines organisation-wide belief systems surrounding the importance of cooperating with competitors (Crick & Crick, 2019). It includes 6 self-rated items presented with a 7-point Likert scale (1 = Very strongly disagree; 7 = Very strongly agree). A school-based vignette was created and questions were slightly modified to fit current study design. Sample items include: “my art club should assist other clubs,” “my art club believes that all rival clubs should cooperate with one another.”

#### Social Desirability Scale

This is a short form of the Marlowe-Crowne Social Desirability Scale developed by Reynolds (1982). It includes 13 self-rated items (Form C) from the original 33-item scale. Participants read each item and decide whether the statement is true (T) or false (F). Sample items include: “I’m always willing to admit to it when I make a mistake,” “I have never deliberately said something that hurt someone’s feelings.”

#### Empathy Quotient (EQ-22)

This is a 22-item Empathy Quotient scale of the original 60-item Baron-Cohen & Wheelwright (2004) version (Wakabayashi et al., 2006). Items are rated on a 4-point Likert scale (1 = Strongly disagree; 4 = Strongly agree). Sample items include: “I am good at predicting how someone will feel,” “I ﬁnd it hard to know what to do in a social situation” (reverse-scored).

#### Big Five Inventory (10-item version)

This 10-item questionnaire is aimed to examine the individual’s personality in five domains: extraversion, agreeableness, conscientiousness, neuroticism, and openness (Rammstedt & John, 2007). Items are rated on a 5-point Likert scale (1 = Disagree strongly; 5 = Agree strongly). Sample items include: “I see myself as someone who is reserved” (extraversion; reverse-scored), “I see myself as someone who gets nervous easily” (neuroticism).

#### Curiosity and Exploration

This is a 10-item questionnaire with two subscales (Kashdan et al., 2009): one subscale measures motivation to seek out new knowledge and new experiences (stretching) and the other subscale measures a willingness to embrace novel, uncertain, and unpredictable nature of everyday life (embracing). Items are rated on a 5-point Likert scale (1 = Very slightly or not at all; 7 = Extremely). Sample items include: “I actively seek as much information as I can in new situations” (stretching), “I prefer jobs that are excitingly unpredictable” (embracing).

#### Creative Self-Efficacy

This is a 6-item questionnaire measuring one’s beliefs that they have the capacity to be creative (Karwowski, 2012). It is a subscale of the Short Scale for Creative Self (SSCS; Karwowski, Lebuda, & Wiśniewska, 2012). Items are rated on a 5-point Likert scale (1 = Definitely not; 5 = Definitely yes). Sample items include: “I know I can efficiently solve even complicated problems,” “I trust my creative abilities.”

#### Grit

This questionnaire measures persistence and passion individuals have to achieve their goals (especially long-term ones; Duckworth & Quinn, 2009). This is a shorter version with 8-items rated on a 5-point Likert scale (1 = Not like me at all; 5 = Very much like me). Scores of 2 dimensions (consistency of interest & perseverance of effort) as well as a total grit score can be obtained. Sample items include: “New ideas and projects sometimes distract me from previous ones.” (consistency of interest; reverse-scored), “I finish whatever I begin” (perseverance of effort).

#### Implicit Theory of Intelligence

This is a 4-item questionnaire measuring an individual’s implicit theory of intelligence, i.e., whether intelligence is fixed or can be changed (Schroder et al., 2015). All items were scored on a Liker scale (1 = Strongly disagree; 6 = Strongly agree). Sample items include: “You have a certain amount of intelligence and you really cannot do much to change it,” “To be honest, you cannot really change how intelligent you are.”

### Social Decision-Making Tasks

The following social decision-making tasks will also be administered along with the socio-cognitive questionnaires. Participants can earn points in these tasks and one round will be randomly selected for each task and the points they earn in that round will be translated to extra payment. In some of the tasks, the participants are told that they will be randomly matched with anonymous partners, who are also adults living in Singapore and participating in this study or other similar studies. Actually, the responses of these partners are pre-recorded choices from an independent group of participants (n = 20) who made choices in similar tasks.

#### Social Value Orientation

This task is adapted from the Slider Measure of Social Value Orientation (SVO; Murphy et al. 2011) and intended to measure an individual’s preferred allocation of resources between him/herself and an anonymous partner (called “Player B”). There are six rounds, with each consisting of 9 options. Each option is in the form of two bars, the height of which represents the points allocated to the participant (“You”) and Player B. For example, in one of the rounds, option 1 has 85 points for “You” and 85 points for Player B; option 9 has 85 points for “You” and 15 points for Player B. Participants were instructed to choose the option they preferred the most. After finishing the 6 rounds, a participant’s SVO value can be derived by calculating the inverse tangent of the ratio between the mean allocations for Player B and the participant (Murphy et al., 2011).


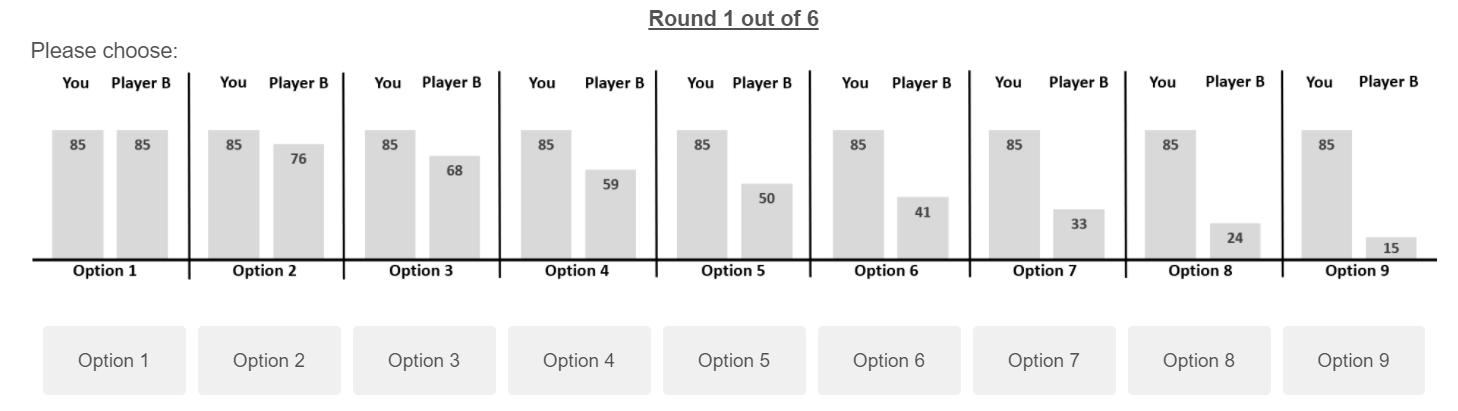


Figure B2: Sample trial from the Social Value Orientation Task

#### Prisoner’s Dilemma, Stag Hunt, and Battle of Sexes

These three games have the same format: A participant and an anonymous partner choose between “RED” and “BLUE”. There are four possible cases: both choosing “RED”; both choosing “BLUE”; the participant choosing “RED” and the partner choosing “BLUE”; and the participant choosing “BLUE” and the partner choosing “RED”. The points they win or lose differ for the four cases. For example, in the prisoner’s dilemma game, when both choose “RED”, both the participant and the partner win 3 points, but when the participant chooses “RED” and the partner chooses “BLUE”, the participant loses 6 points and the partner wins 6 points. The points the players win or lose in the four cases are different for the three games. Each game consists of 1 practice round and five formal rounds. Participants play with a different partner for each round. A participant’s cooperativeness can be measured by the proportion of cooperative choices in the five formal rounds.


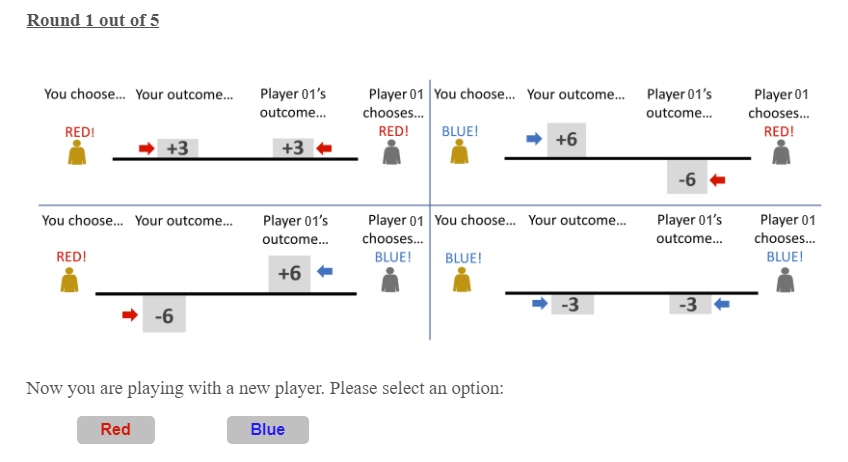


Figure B3: Sample trial from the Prisoner’s Dilemma Game

#### Risk Preference (positive, negative, and mixed domain)

These three tasks are adapted from Bohnet et al., (2008). In each task, risk preference is measured with the multiple price list paradigm (Holt & Laury, 2002) in which a participant makes nine binary choices between a sure option and a risky option. In the positive domain, the sure option gives 100% chance of earning 10 points; the probabilities for the risky option range from 90% chance of earning 8 points and 10% chance of earning 15 points to 10% chance of earning 8 points and 90% chance of earning 15 points across the nine rounds. In the negative domain, the sure option gives 100% chance of losing 10 points; the probabilities for the risky option range from 90% chance of losing 8 points and 10% chance of losing 15 points to 10% chance of losing 8 points and 90% chance of losing 15 points across the nine rounds. In the mixed domain, the sure option gives 100% chance of not earning or losing any points; the probabilities for the risky option range from 90% chance of earning 5 points and 10% chance of losing 2 points to 10% chance of earning 5 points and 90% chance of losing 2 points across the nine rounds. In tasks applying the multiple price list paradigm, the round where a participant switches over from the sure option to the risky option (or vice versa) can be used to indicate the participant’s risk preference. Logistic regression will be used to derive the switch point (Engel & Kirchkamp, 2019).


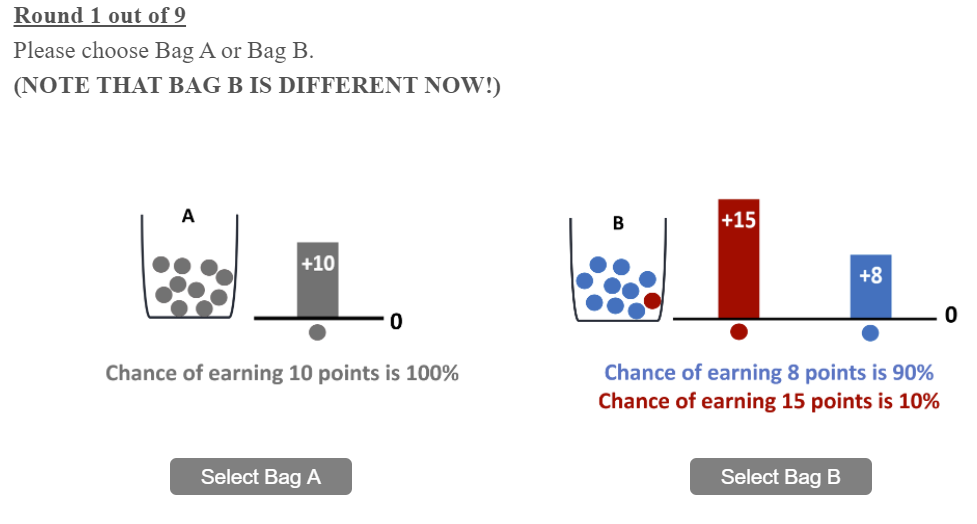


Figure B4: Sample trial from the Risk Preference Task (positive domain)

#### Ambiguity Aversion

Ambiguity aversion is measured with the multiple price list paradigm (Holt & Laury, 2002) in which a participant makes nine binary choices between a risky option and an ambiguous option. Across the nine rounds, the probabilities for the risky option range from 90% chance of earning 15 points and 10% chance of earning 8 points to 10% chance of earning 15 points and 90% chance of earning 8 points; the ambiguous option shows at least 10% chance of earning 15 points and at least 10% chance of earning 8 points without revealing information of the other 80% probability. As a measure of ambiguity aversion, the switch point from one option to the other will be derived by logistic regression (Engel & Kirchkamp, 2019).


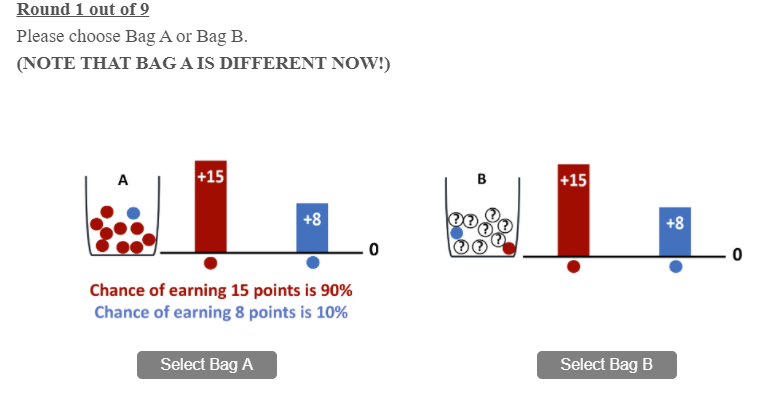


Figure B5: Sample trial from the Ambiguity Aversion Task

#### Risky Dictator

This task is adapted from Bohnet et al., (2008). It measures the risk preference when the payoffs go to both the participant and an anonymous partner. It also applies the multiple price list paradigm (Holt & Laury, 2002). A participant makes 10 choices between a sure option and a risky option. In the sure option, the participant gains 10 points and the partner gains 10 points; in the risky option, the probabilities range from 10% chance of 15 points for the participant and 15 points for the partner and 90% chance of 8 points for the participant and 22 points for the partner to 100% chance of 15 points for the participant and 15 points for the partner and 0% chance of 8 points for the participant and 22 points for the partner across the 10 rounds. For the risky option, an image of a fortune wheel with different proportions of red and green sections is used to indicate the probabilities of the two possible outcomes. This is also to show that the outcomes are determined by nature rather than the partner’s decision. The participants play with a different partner in each round. As a measure of risk preference, the switch point from one option to the other will be derived by logistic regression (Engel & Kirchkamp, 2019).
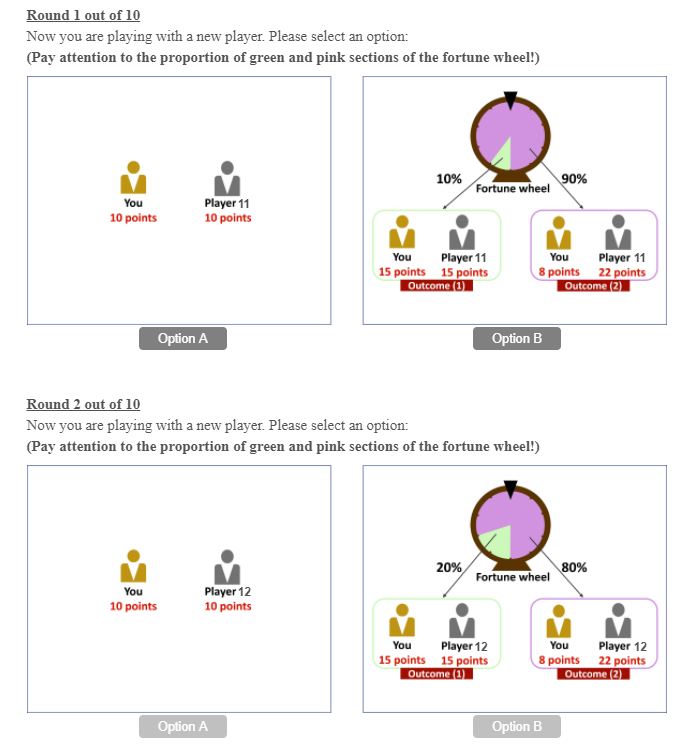


Figure B6: Sample trial from the Risky Dictator Game

#### Trust Game (Without History, With History, Participants as Player B)

These tasks are also adapted from Bohnet et al., (2008). The “without history” and “with history” versions measure a participant’s trust in an anonymous partner, which is the belief that this partner will allocate points fairly between the participant and the partner. The “without history” version consists of five rounds. In each round, a participant chooses between a sure option and a risky option. If the sure option is chosen, the participant gains 10 points and the partner gains 10 points. If the risky option is chosen, the partner will choose between the two outcomes: 15 points for the participant and 15 points for the partner and 8 points for the participant and 22 points for the partner. There is no information about the partner’s past choices and in each round, the participants play with a different partner. In this “without history” version, a participant’s trust can be measured by the proportion of trusting choices (choosing the risky option) in the five rounds.

In the “with history” version, we also applied the multiple price list paradigm (Holt & Laury, 2002) and a participant makes 10 choices between a sure option and a risky option. If the sure option is chosen, the participant gains 10 points and the partner gains 10 points. If the risky option is chosen, the partner will choose between the two outcomes mentioned above in the “without history” version. However, the partner’s past choices between these two outcomes will be shown to the participant. A pie chart with different proportions of red and green sections is used to indicate the frequencies of the partner choosing the two outcomes in the past. For the risky option, the frequencies of the partner’s choice history range from 10% chance of 15 points for the participant and 15 points for the partner and 90% chance of 8 points for the participant and 22 points for the partner to 100% chance of 15 points for the participant and 15 points for the partner and 0% chance of 8 points for the participant and 22 points for the partner across the 10 rounds. The participants play with a different partner in each round. As a measure of trust, the switch point from one option to the other will be derived by logistic regression (Engel & Kirchkamp, 2019).


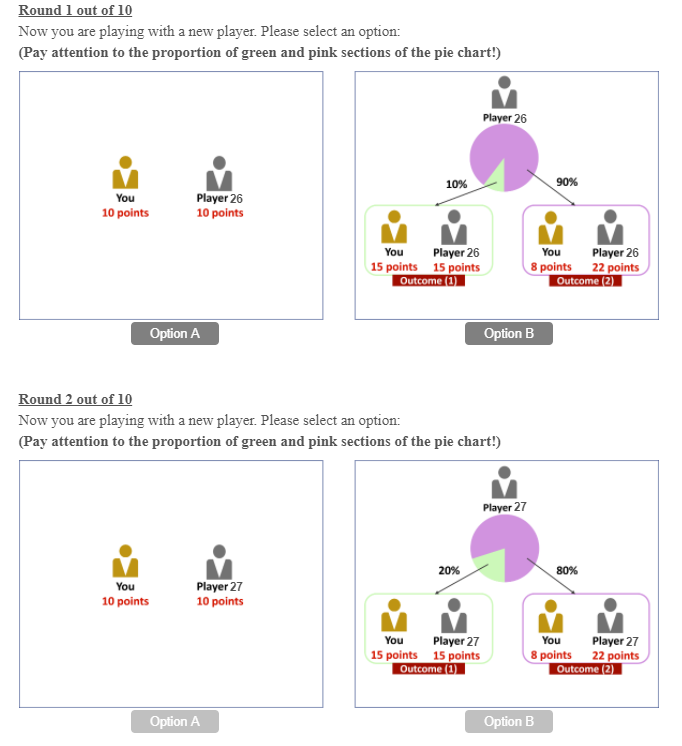


Figure B7: Sample trial from the Trust Game (with history)

In the “participants as Player B” version, the participants are in the role of making choices between the two outcomes: 15 points for the participant and 15 points for the partner and 22 points for the participant and 8 points for the partner. Note that the points for the two players are also switched as the participants have a chance to gain 22 points. This version consists of 5 rounds and the participants play with a different partner in each round. A participant’s trustworthiness can be measured with the proportion of choosing the fair outcome (15 points for both players) in the five rounds.


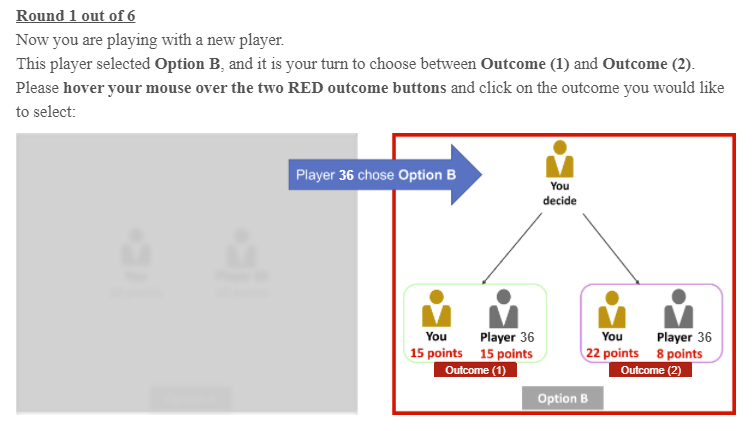


Figure B8: Sample trial from the Trust Game (as Player B)

## S3 Appendix C. Technical Screening Questionnaire for Remote Guided Testing (RGT) eligibility

| 1. Participant ID: ____________ |  |
| --- | --- |
| 1. My computer is a:    1. Laptop    2. Desktop    3. Others: ______ |  |
| 1. The brand of my computer is:    1. Apple    2. HP    3. Lenovo    4. Dell    5. Acer    6. Fujitsu    7. Asus    8. Others: ______ |  |
| 1. The processor in my computer is:    1. Intel Core i3    2. Intel Core i5    3. Intel Core i7    4. Intel Core i9    5. Apple M1    6. Apple M2    7. Others: ______ | Minimally Intel Core i3 |
| 1. My computer RAM is:    1. 8GB    2. 16GB    3. 32GB    4. Others: ______ | Minimum 8GB |
| 1. My computer's free hard disk space is:    1. 256GB    2. 512GB    3. 1TB    4. Others: ______ | Minimally 10GB |
| 1. My computer's total hard disk space is:    1. 256GB    2. 512GB    3. 1TB    4. Others: ______ |  |
| 1. My physical display screen size in inches, measured diagonally is (Please only state the numerical value of the screen size - e.g., 13) | Screen height minimally 15cm, 12-inch diagonally with 16:9 is a minimum |
| 1. My computer's screen refresh rate in Hertz is (Please only state the numerical value of the refresh rate - e.g., 60) | Minimally 60 |
| 1. My computer's screen resolution in pixels is (Please only state the numerical value of your screen resolution - e.g., 2560x1600) | Minimally 1366x768 |
| 1. My mouse is:    1. Wired (please state the brand and model)    2. Wireless (please state the brand and model) | Wired |
| 1. My keyboard is:    1. Integrated    2. Wired (please state the brand and model)    3. Wireless (please state the brand and model)    4. Others: ______ | Integrated or wired |
| 1. My webcam is:    1. Integrated    2. Separate Device (please state the brand and model)    3. Others: ______ |  |
| 1. My microphone is:    1. Integrated    2. Separate Device (please state the brand and model)    3. Others: ______ |  |
| 1. My earpiece is:    1. Wired (please state the brand and model)    2. Wireless (please state the brand and model) | Wired |
| 1. My operating system and the version operating system is (e.g., MacOS Mojave Version 10.14.6) | Windows or MacOS |
| 1. My web browser is:    1. Google Chrome (Recommended)    2. Safari    3. Mozilla Firefox    4. Others: ______ |  |
| 1. Please select the range of your download speed (you can check this at:  <https://speed.measurementlab.net/#/>)    1. 0-5mb/s    2. 6-10mb/s    3. Others: ______ |  |
| 1. Please select the range of your upload speed (you can check this at:  <https://speed.measurementlab.net/#/>)    1. 0-5mb/s    2. 6-10mb/s    3. Others: ______ |  |
| 1. Please select the range of your latency (you can check this at:   <https://speed.measurementlab.net/#/>)   - 1. 0-25ms   2. 26-50ms   3. 51-75ms   4. 76ms-100ms   5. Others: ______ |  |
| 1. Any additional details that you would like to add about your hardware device |  |

## S4 Appendix D. Details of Cognitive task battery

### Wisconsin Card Sorting Task

We use a computerised version of Wisconsin Card Sorting Task (Grant & Berg, 1948), implemented on the Inquisit platform (Inquisit 6, 2019). Participants are asked to sort the card into four different categories. Participants receive no information regarding the sorting rules and need to figure out the rules based on the feedback they get after each card sort. The maximal number of trials is 128. The variables of interest are the number of perseverative errors and computational modelling parameters (learning rate, inverse temperature) from reinforcement learning models.


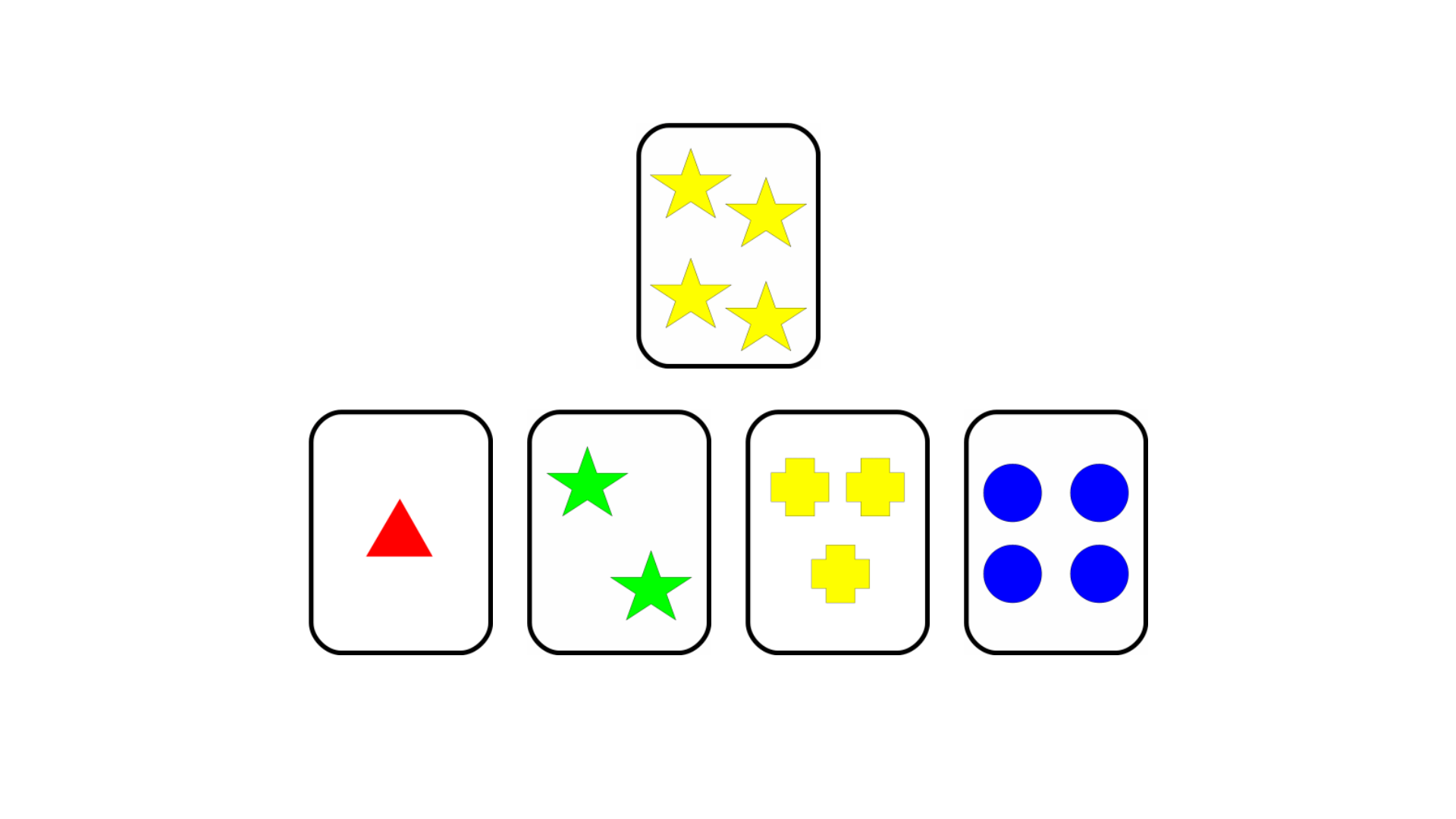


Figure D1: Sample trial from the Wisconsin Card Sorting Task

### Probabilistic Reversal Learning

We use a computerised version of Probabilistic Reversal Learning (PRL) task (Cools et al., 2002), implemented on the iABC platform (iabc.psychol.cam.ac.uk/welcome). In the PRL task, participants are presented with 2 different coloured patterns. In the first phase (the “discrimination phase”, trial 1 to 40), they select one of the two patterns and are informed of whether their decision was “correct” or “incorrect”. The chances of showing accurate feedback are probabilistic (e.g., yellow patterns will show accurate feedback 80% of the time, blue 20%). In the second phase, these probabilities are reversed (the “reversal phase”, trial 41 to 80). Cognitive flexibility is measured from an examination of how well the participant adjusted to the new probabilistic conditions. The variables of interest are the number of perseverative errors and computational modelling parameters (learning rate, inverse temperature) from reinforcement learning models.


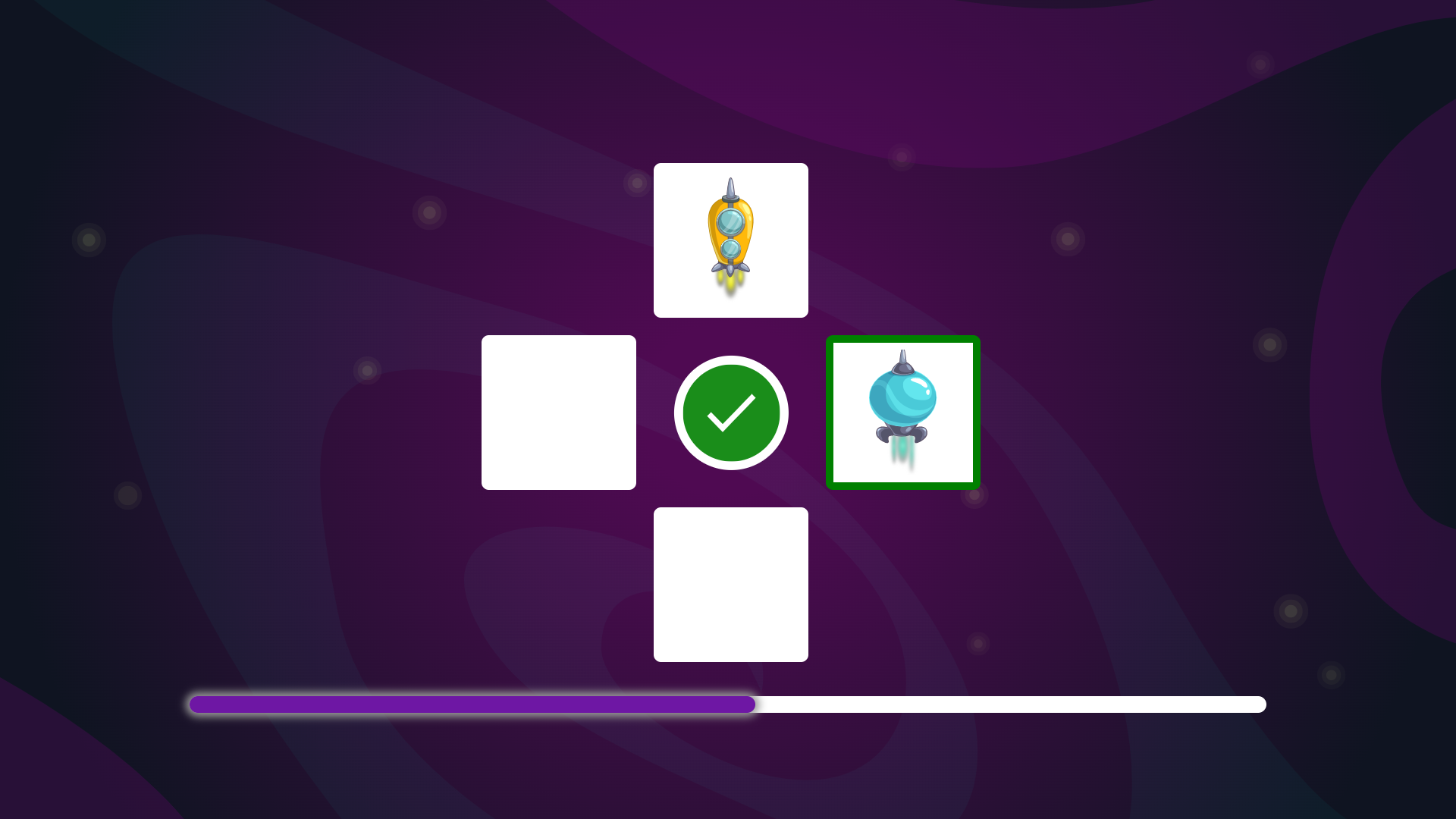


Figure D2: Sample Probabilistic Reversal Learning trial, with feedback indicating that pattern selection was correct.

### Intra-/Extra-Dimensional Set Shifting Task

The CANTAB Intra-/Extra-Dimensional Set Shifting (IED) task tests participants’ ability in visual discrimination, attentional set formation maintenance, as well as attention shifting and flexibility (Robbins et al., 1998). Participants must use feedback to develop a rule that determines whether stimulus is correct in this task. IED begins with simple stimuli that consist of only one of the dimensions. Compound stimuli are utilised later in the task. The rule shifts occur intra-dimensionally at first and then extra-dimensionally. The variables of interest will be the number of errors in the extra-dimensional shift stage and computational modelling parameters (learning rate, inverse temperature) from reinforcement learning models.


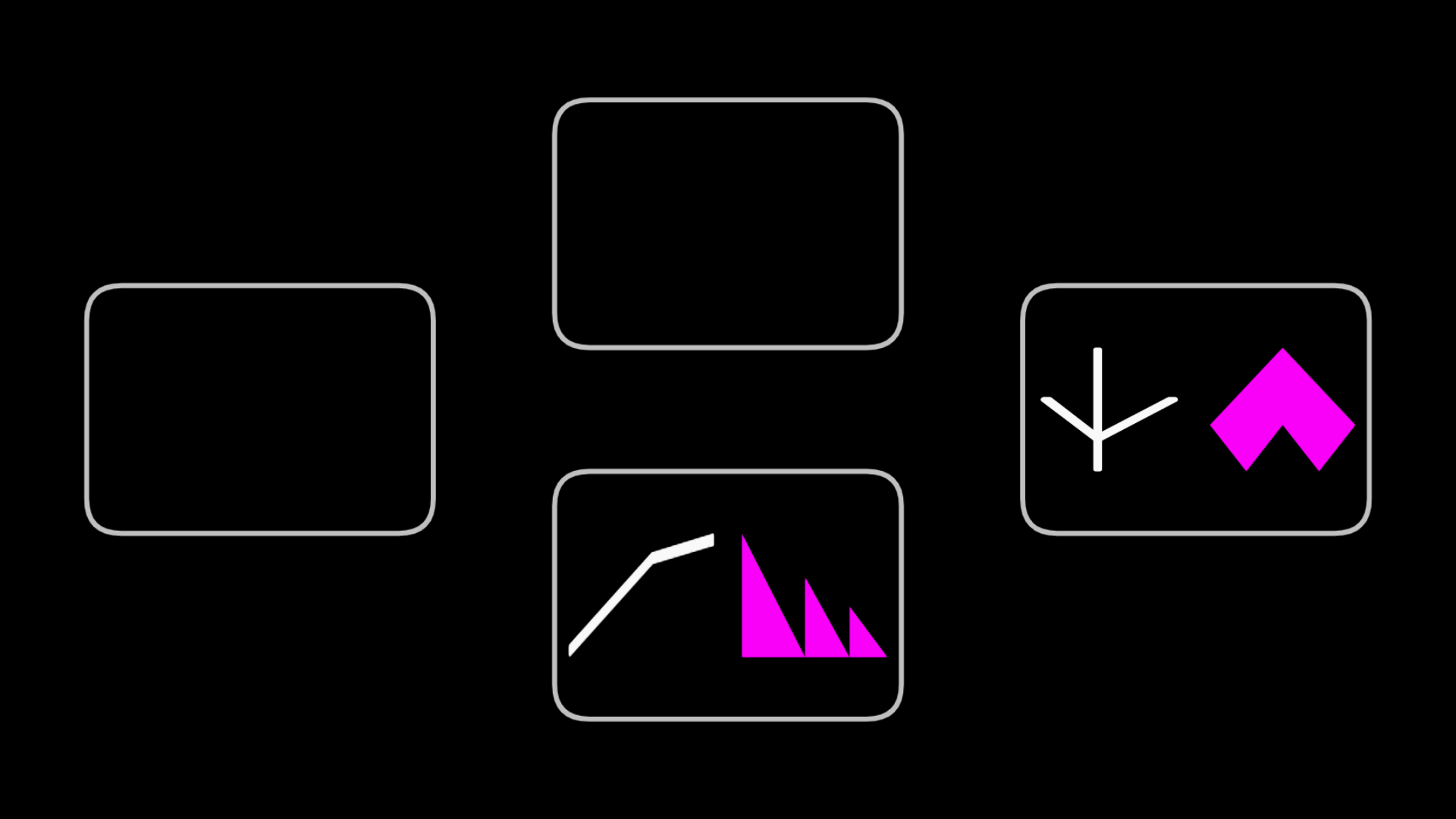


Figure D3: Sample IED trials

### Task Set Switching

Task Set Switching (TSS) assesses participants’ ability to switch between different tasks (Monsell, 2003). We use a computerised task on the Gorilla platform (www.gorilla.sc). On each trial, participants will be presented with one letter and one number (e.g., 8A) in either a circle or a square. Before testing starts, participants will learn the rules of two tasks: when they see a circle, they should indicate whether the letter is on the right or the left side, and when they see a square, they should indicate whether the number is on the right side. Participants will be asked to switch periodically between performing number tasks or letter tasks. The variable of interest will be the switch cost in accuracy and RT (difference between shift-trials and non-shift trials).


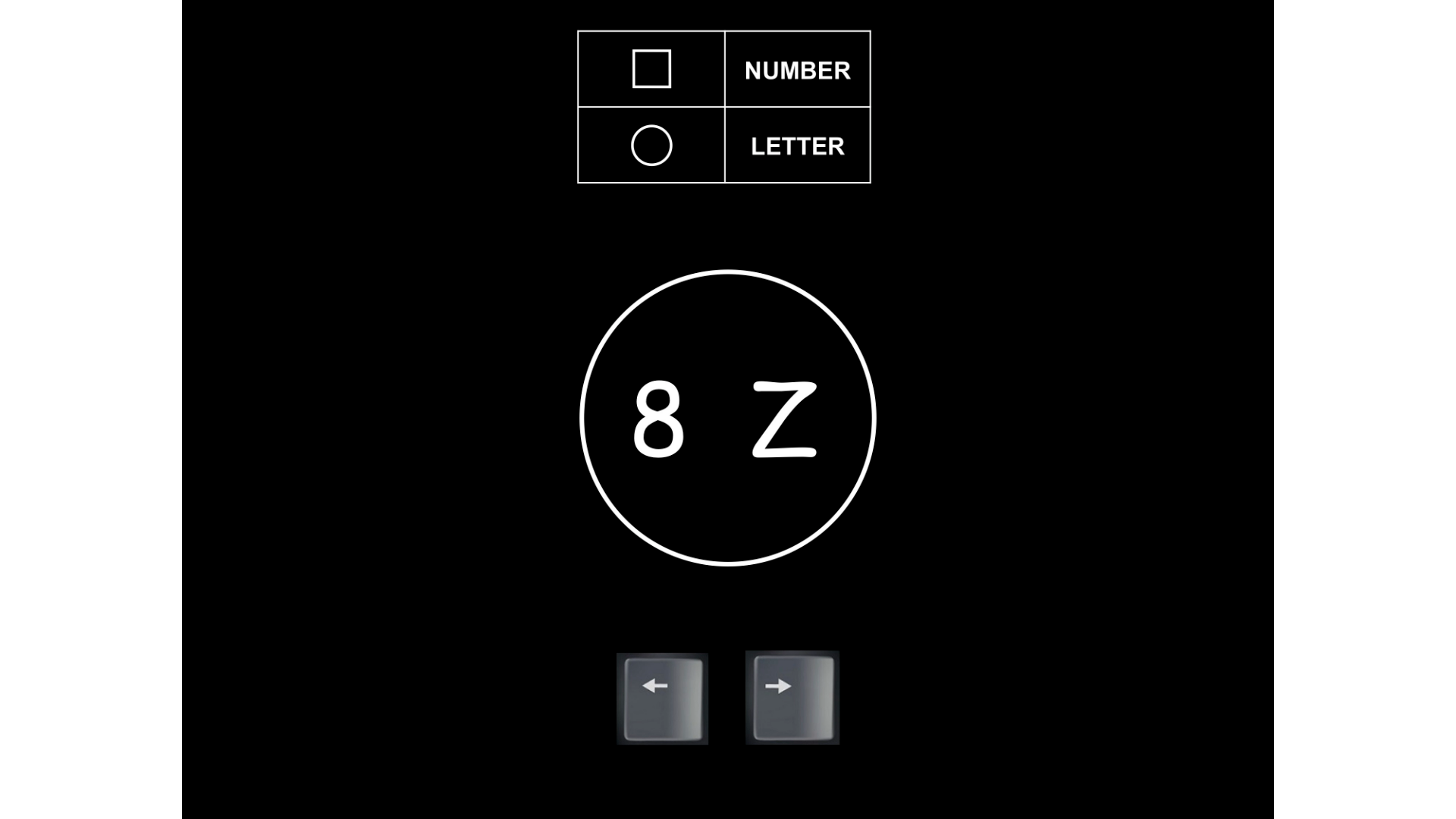

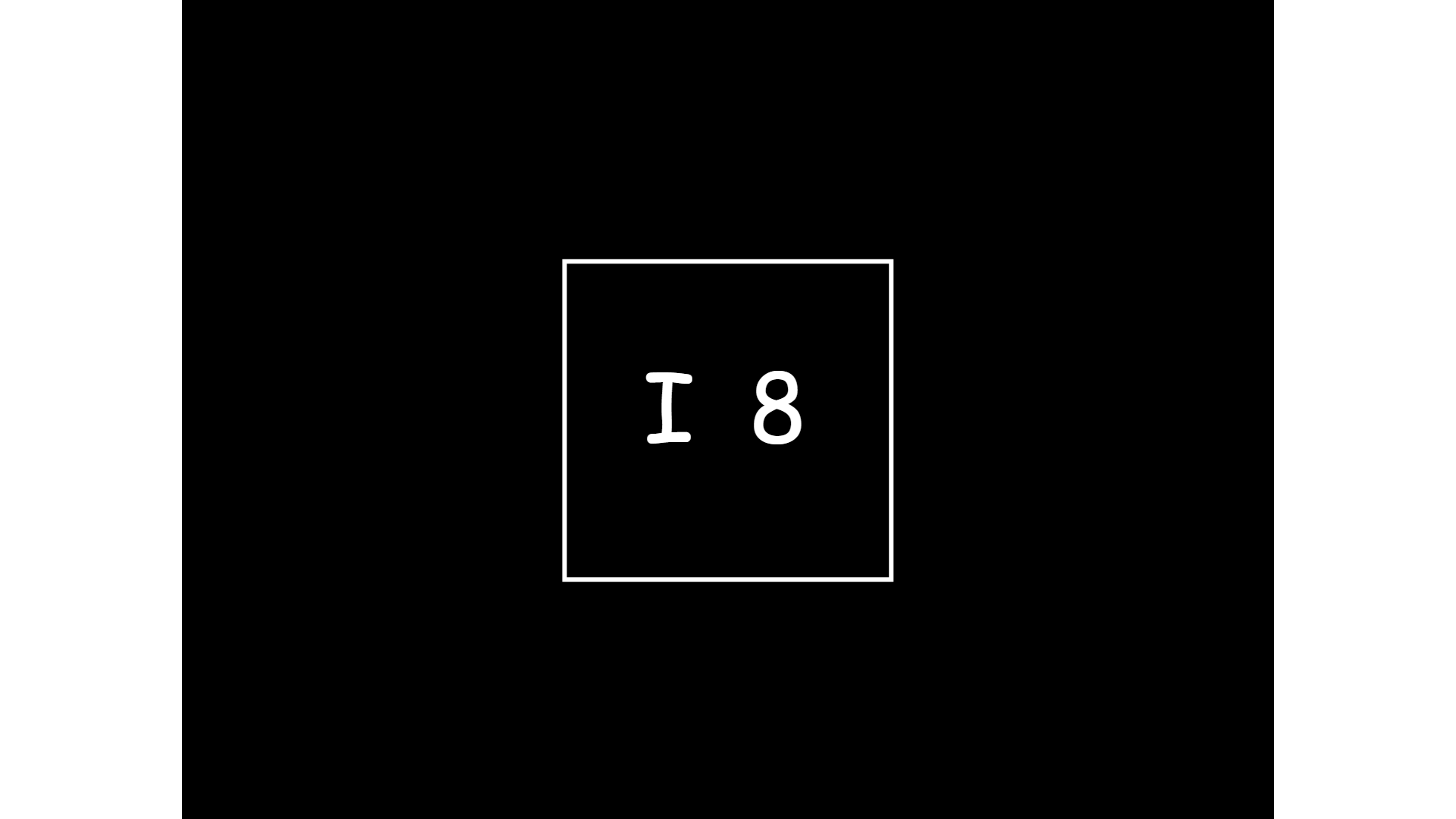


Figure D4: (Left) Sample Task Set Switching Trial during the Practice Session. (Right) Sample Task Set Switching Trial during actual task.

### Trail Making Test

Trail Making Test (TMT) is a neuropsychological test of visual attention and task switching (Reitan, 1958). We use a computerised version of TMT on the Inquisit platform (Inquisit 6, 2019). In this task, participants are asked to draw lines to connect a set of items in sequence as quickly and accurately as possible. In the first trail (trail A), participants need to connect by numbers (e.g., 1-2-3-4…). In the second trail (Trail B), participants need to connect the items by both numbers and letters, switching between the connecting rules by each item (e.g., 1-A-2-B-3-C…). The variable of interest will be the switch cost in errors made and time to complete the trails A and B.


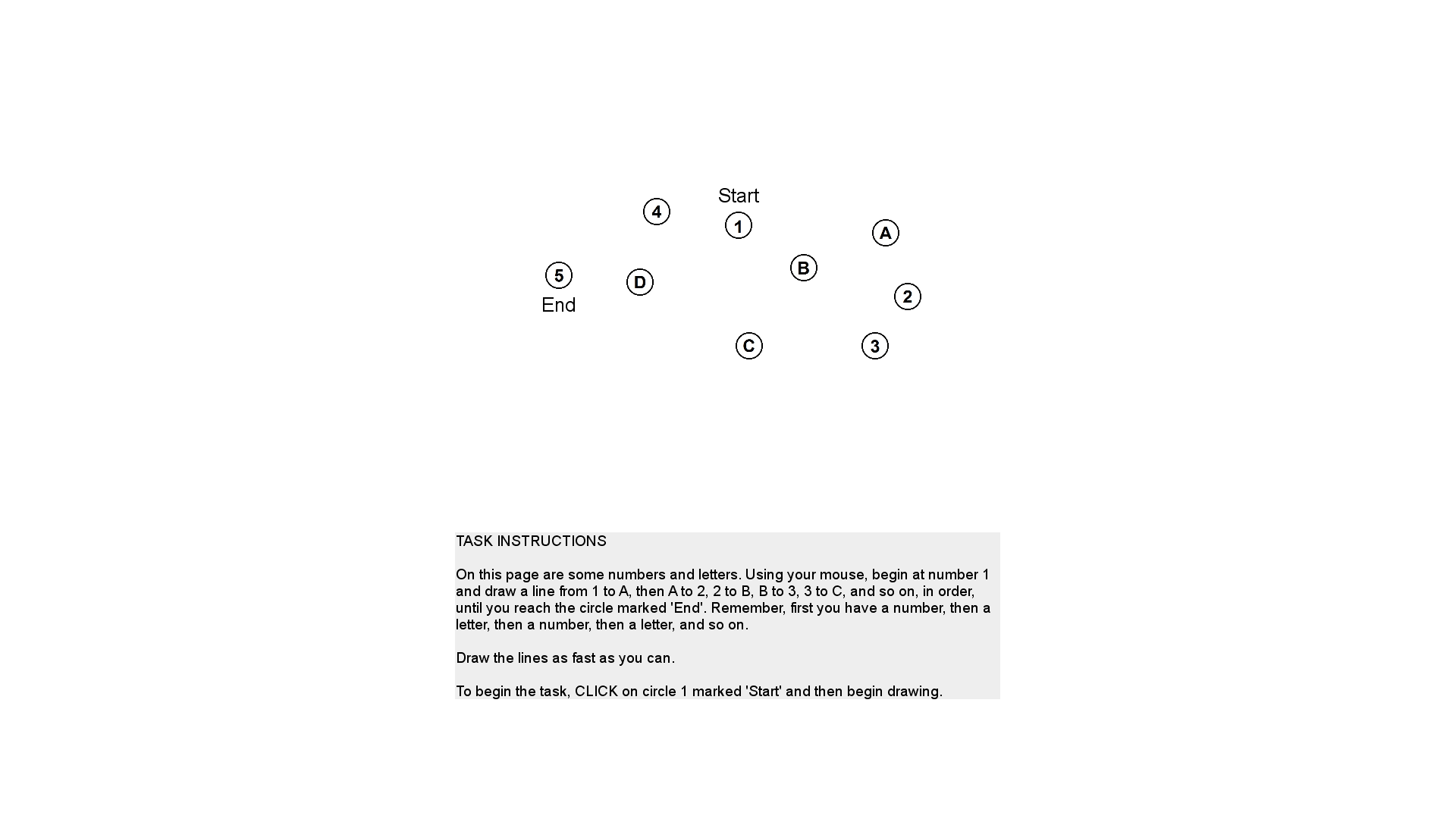


Figure D5: Sample practice trial (Trail B) for Trail making test.

### CANTAB Spatial Working Memory

The CANTAB Spatial Working Memory (SWM) task tests retention and manipulation of visuospatial information (Robbins et al., 1998). SWM starts with a screen filled with various coloured boxes. Participants’ goal is to find all the tokens in the boxes with a minimum number of attempts. Participants need to avoid revisiting boxes that are empty or boxes from which they have collected tokens before. The total number of boxes for the participants to search gradually increases until a maximum of 12 boxes are shown. Total errors (selecting boxes that have already been determined to be empty and revisiting boxes that have already been discovered to contain a token) and strategy scores are the two outcome measures.


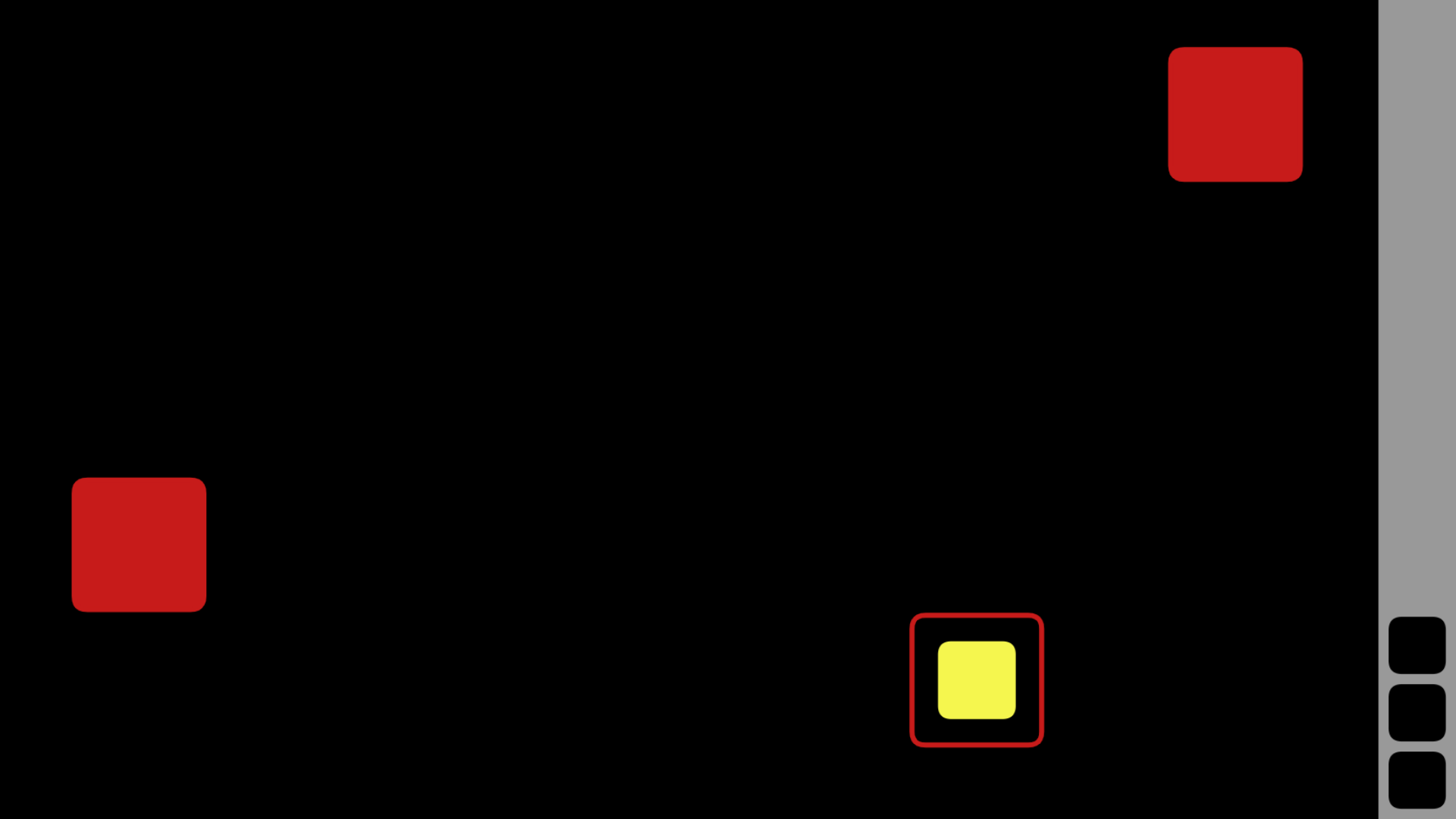


Figure D6: Sample trial of the Spatial Working Memory task

### Backwards Digit Span

In Backwards Digit Span (BDS), participants will hear a series of numbers such as “4-1-3-6”. Participants are asked to verbally repeat the numbers to the experimenter in reversed order (“6-3-1-4”) (Conway et al., 2005). Outcome measure is the total correctly recalled digits. BDS is conducted manually.

### Reading Span

We adapted the reading span (RS) subtest of the Adaptive Composite Complex Span Test (ACCES) (Gonthier et al., 2016). RS is conducted on the Inquisit platform (Inquisit 6, 2019). The sentence judging materials were replaced with longer sentences for the adult study. On each trial, participants will first see alternating sentences and single-digit numbers. The task is to judge whether the sentences make sense, while remembering the numbers in order. By the end of the sentence-number sequence, participants are asked to recall the number sequence using a number pad. Each participant completed six trials. The length of the number sequences varies between 3 to 8 and is delivered adaptively to match the participant’s performance. Outcome variable will be the total number of correctly recalled digits.


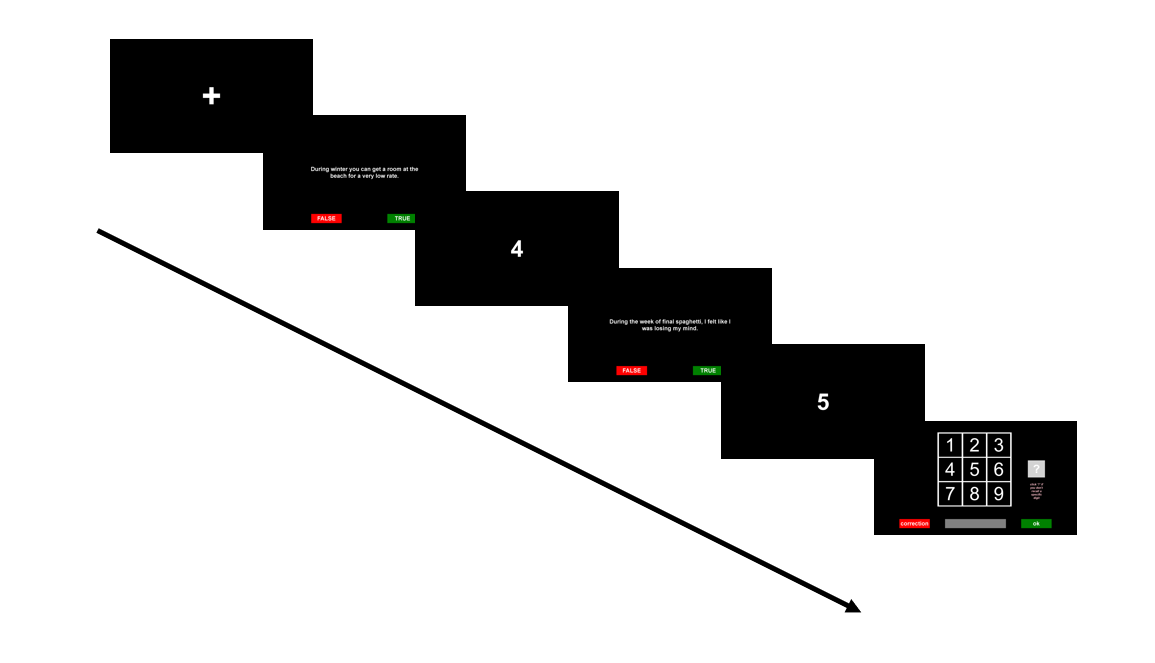


Figure D7: Sample trial of the Reading Span task.

### Stop Signal Task

We use a computerised version of the Stop Signal Task (SST) (Verbruggen & Logan, 2009) on the Inquisit platform (*Inquisit 6*, 2021) to assess the ability to inhibit a response. On each trial, participants are presented with an arrow on the screen. Participants are asked to indicate the direction in which it is pointing as quickly as possible. However, on some STOP trials, participants will hear a beep, indicating that they should not respond on that trial. We will follow the recommendation of (Verbruggen et al., 2019) to estimate the stop signal reaction time, which is our variable of interest.


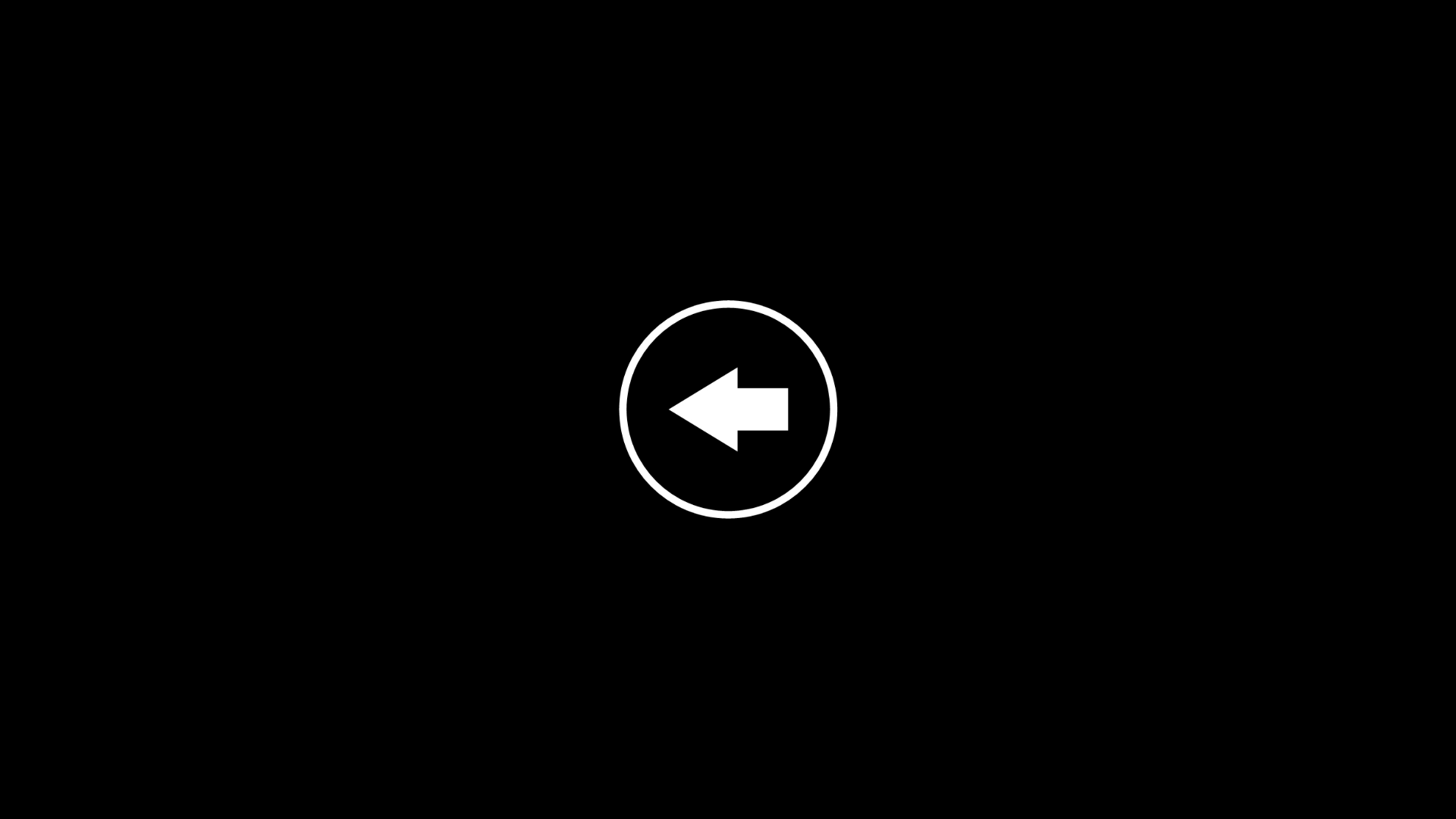


Figure D9: Sample SST trial with arrow pointing to the left.

### Stroop Task

We use a computerised version of the Stroop test (Stroop, 1935) on the Inquisit platform (*Inquisit 6*, 2021). This task is a test of impulse control, typically using a colour-word task. Participants will see a colour word on the screen, but the print colour may or may not match the meaning of the word (e.g., “RED” printed in the colour red, or green). Participants are instructed to ignore the meaning of the word and only indicate the print colour of the word as quickly as possible. Each participant completed 180 trials. The variables of interest will be the switch cost of accuracy and RT between congruent and incongruent trials.


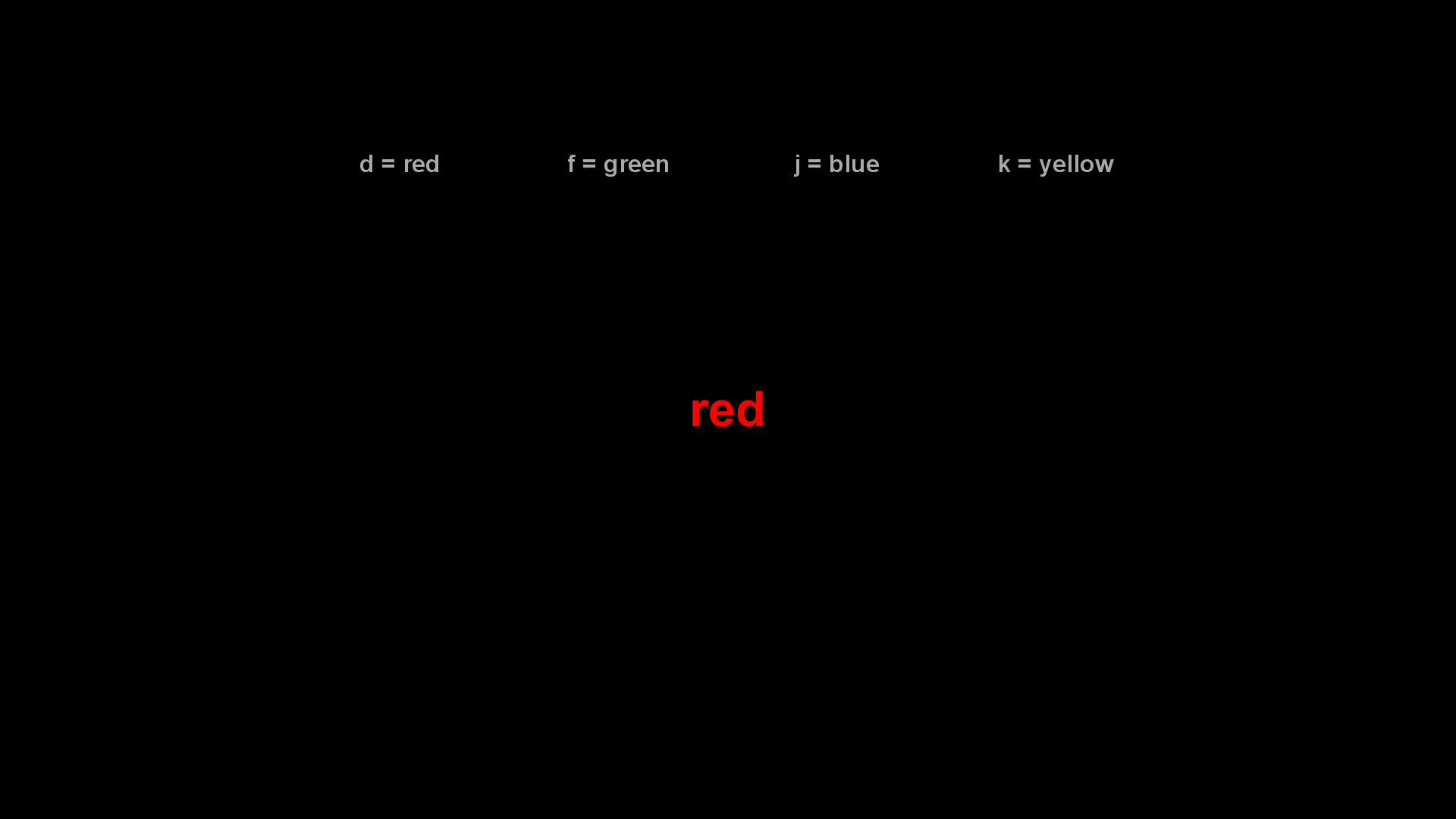

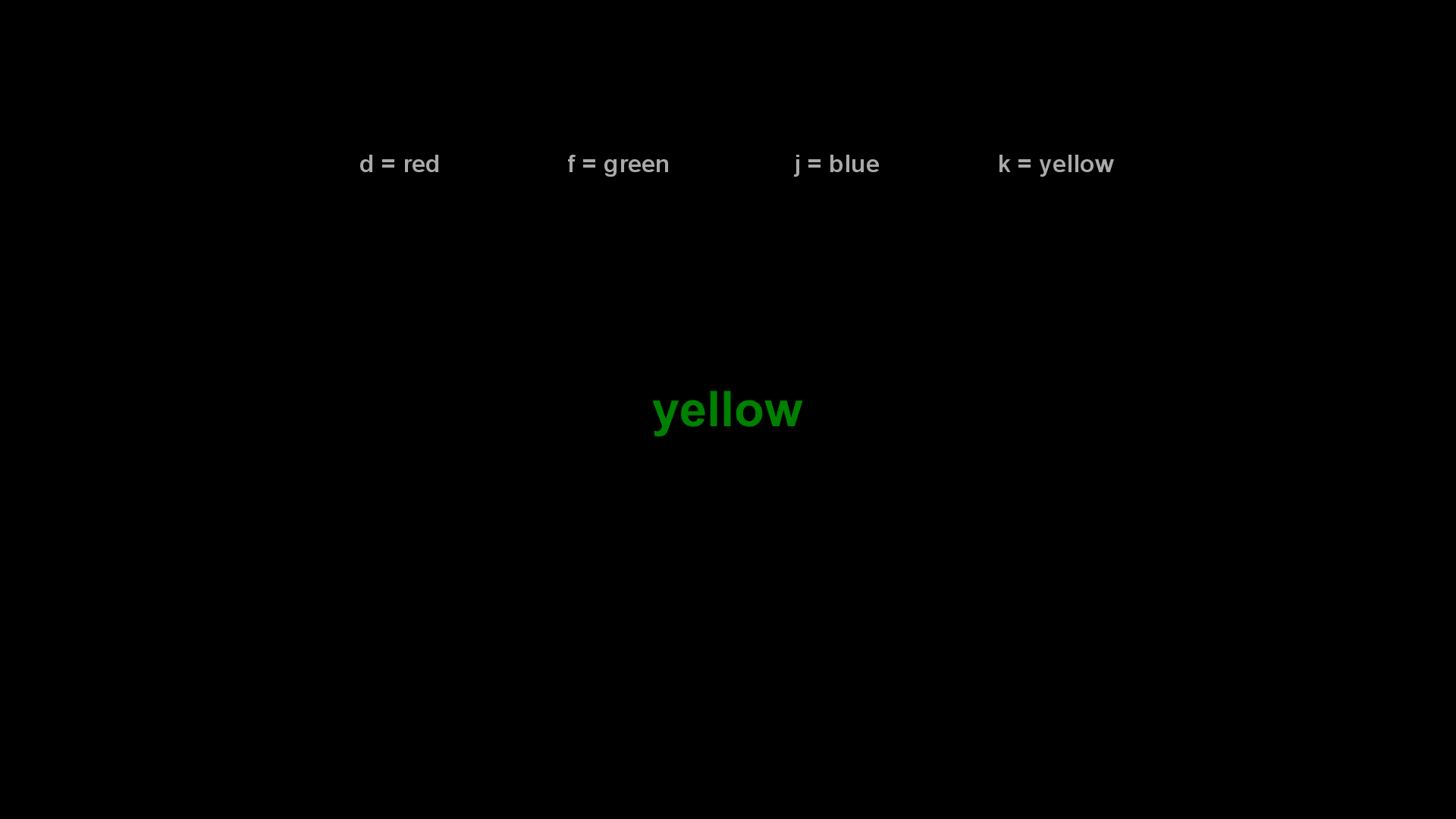


Figure D10: Sample trial containing congruent (left) and incongruent (right) visual stimuli

### Structure Learning

Structure learning (SL) involves making predictions based on previously learned patterns in the stimuli (Wang et al., 2017a, 2017b). On each trial, participants will see a sequence of visual symbols. Then, participants will be presented with four symbols and asked to predict which symbol they think should come next in the sequence. Each participant completes three sessions of SL task, on three separate sittings. Each session consists of four blocks and each block consists of 60 trials. No trial level feedback is given, but at the end of each block, participants are presented with a score based on their task performance. SL is conducted on the iABC platform (iabc.psychol.cam.ac.uk/welcome). Outcome variables will be the Performance Index and Strategy scores, detailed in Wang et al. (2017a).


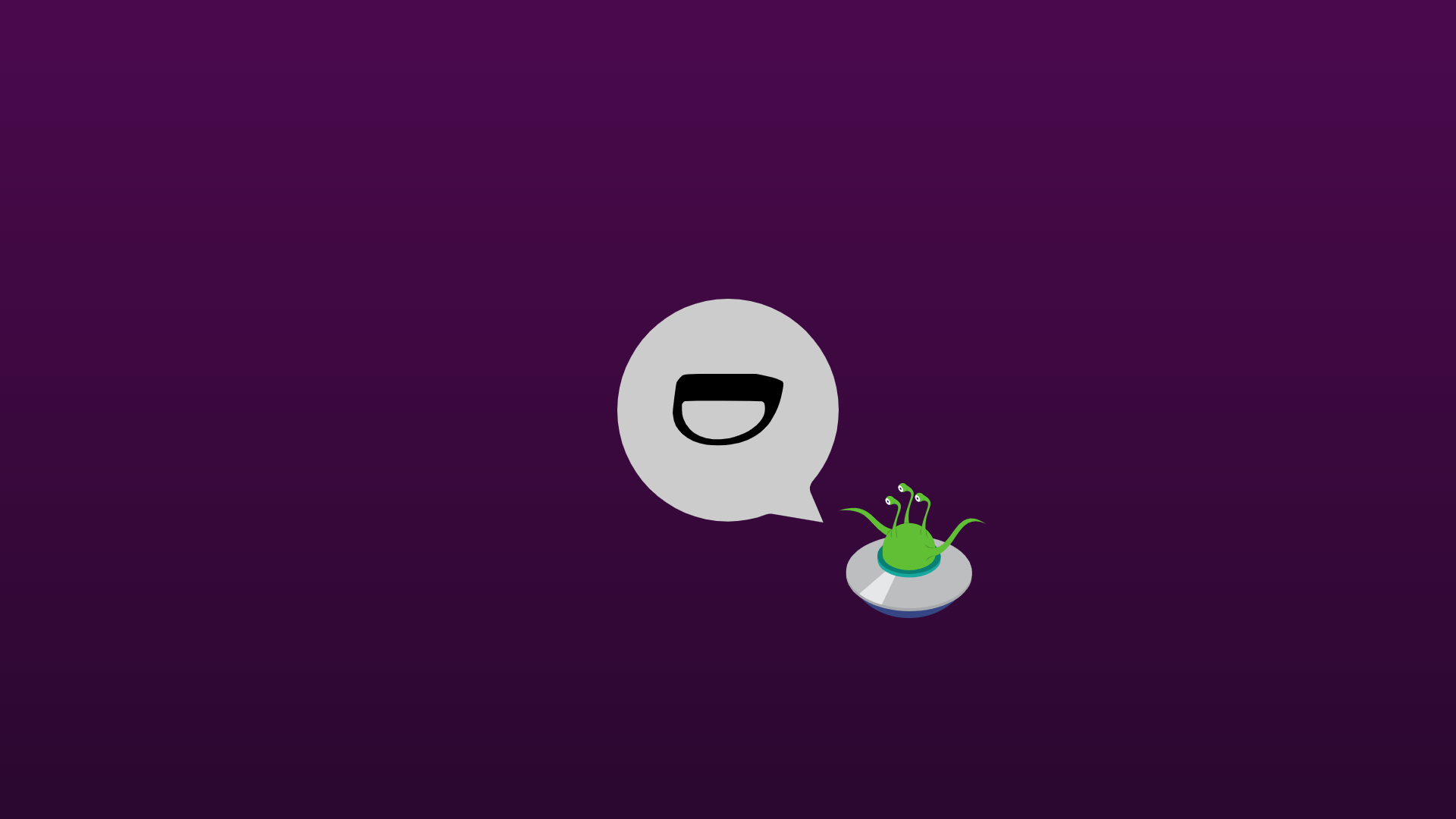

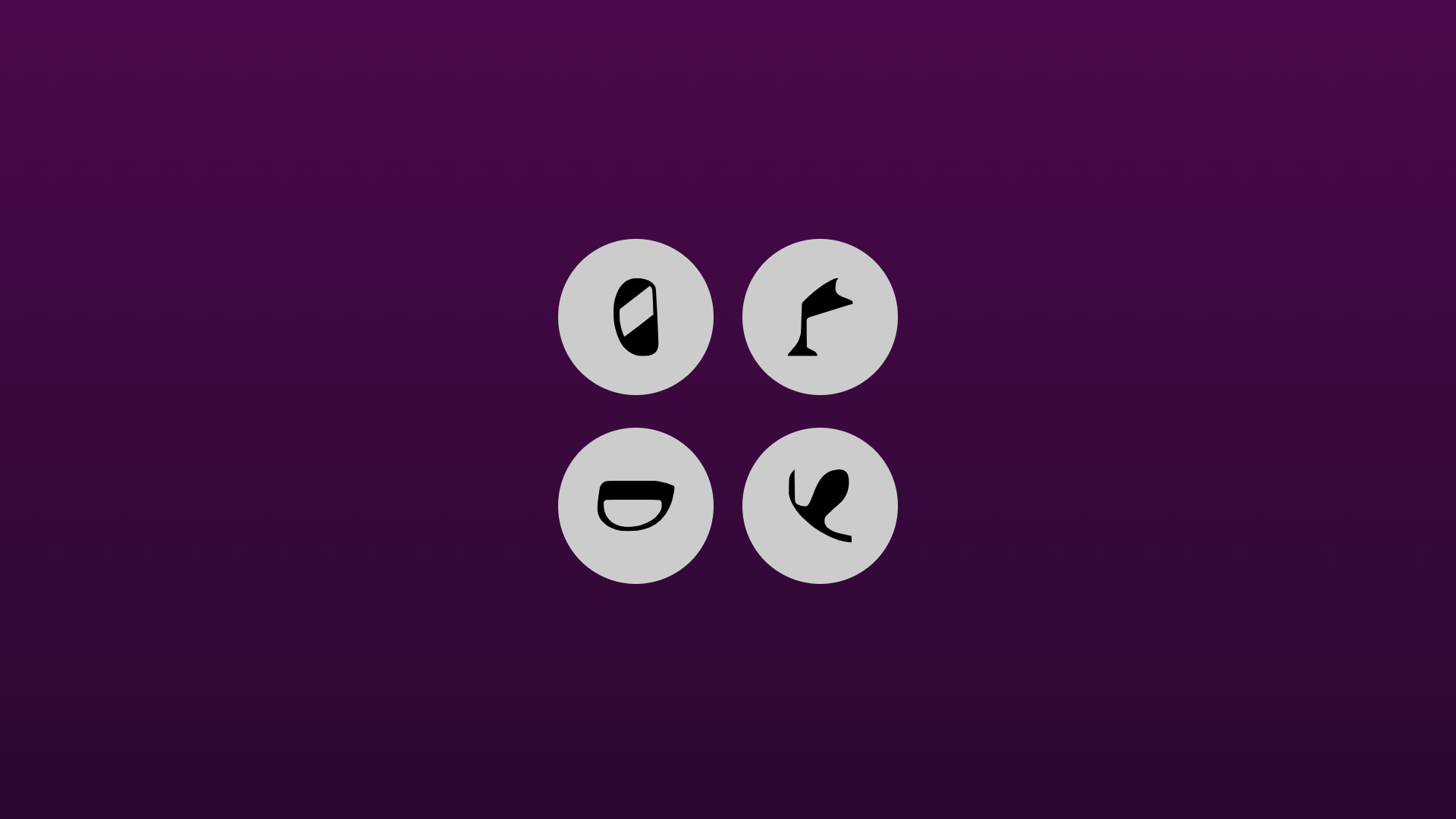


Figure D8: Sample trial of the structure learning task. Left: a sequence of symbols will briefly appear one by one on the screen. Right: participants will be asked to predict the next symbol by choosing from the four symbols shown.

### Alternate Uses Task

We use a computerised version of the AUT (Guilford, 1967) on the Inquisit platform (*Inquisit 6*, 2021). Participants are shown common household items and are asked to generate as many possible but atypical uses for the items. Each participant completes one practice trial (brick) and five test trials (shoe, paperclip, nail, button, newspaper). Participants are given two minutes for each item. The testing screen has a timer to remind participants of how much time they had left to complete the task. The variable of interest is the total fluency, originality, and flexibility scores.


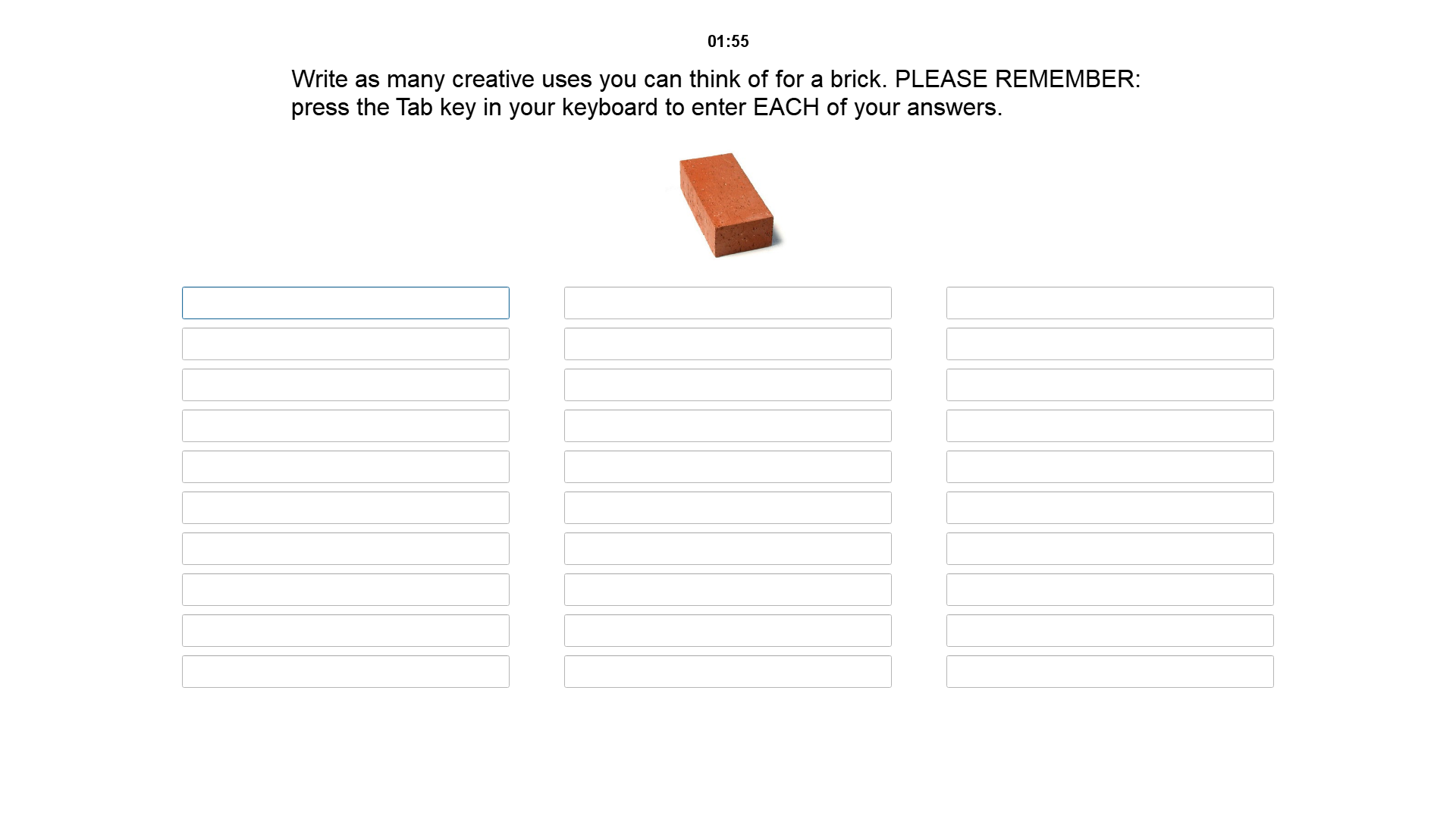


Figure D14: AUT Practice Trial, with a Brick as the object of focus.

### Torrance Test of Creative Thinking - Figural

We use Form A of the standard version of TTCT-F (Torrance, 1990). The task includes three activities: (1) picture construction, (2) picture completion, and (3) addition of lines. In all three activities, participants are asked to complete a picture based on the different stimuli provided and assign a title for the picture completed. Ten minutes were provided to the participants to complete each activity. Participants’ TTCT-F performance is scored following the streamlined scoring guide (Torrance, 1990) The scores of five subscales (fluency, originality, abstractness of titles, elaboration, and resistance to premature closure) will be the outcome variables.

### Verbal Fluency Test

We assessed verbal fluency with letter and category fluency subtasks. For the letter fluency subtask, participants will be instructed to produce as many nouns or verbs as possible starting with the respective letters (F, A and S). Participants will be prompted not to repeat words or say the same words with different endings (e.g., fish, fishing, fisheye etc.). For the category naming subtask, participants will be instructed to say as many items belonging to the animal category within a minute. Time will be limited to a 60 second period for each letter/ category (Lezak et al., 2004). The variables of interest will be the numbers of eligible words generated in each category.

### Remote Associates Test

In each RAT question, participants are presented with three words. Participants are asked to come up with a fourth word related to all three given words and could combine it to a word or common phrase. Each participant completes 30 RAT questions. We adapted the test questions to the Singaporean language context in a pilot study. We use a computerised version of the RAT (Mednick & Mednick, 1967) on the Inquisit platform (*Inquisit 6*, 2021). The variable of interest is the percentage of correct solutions.


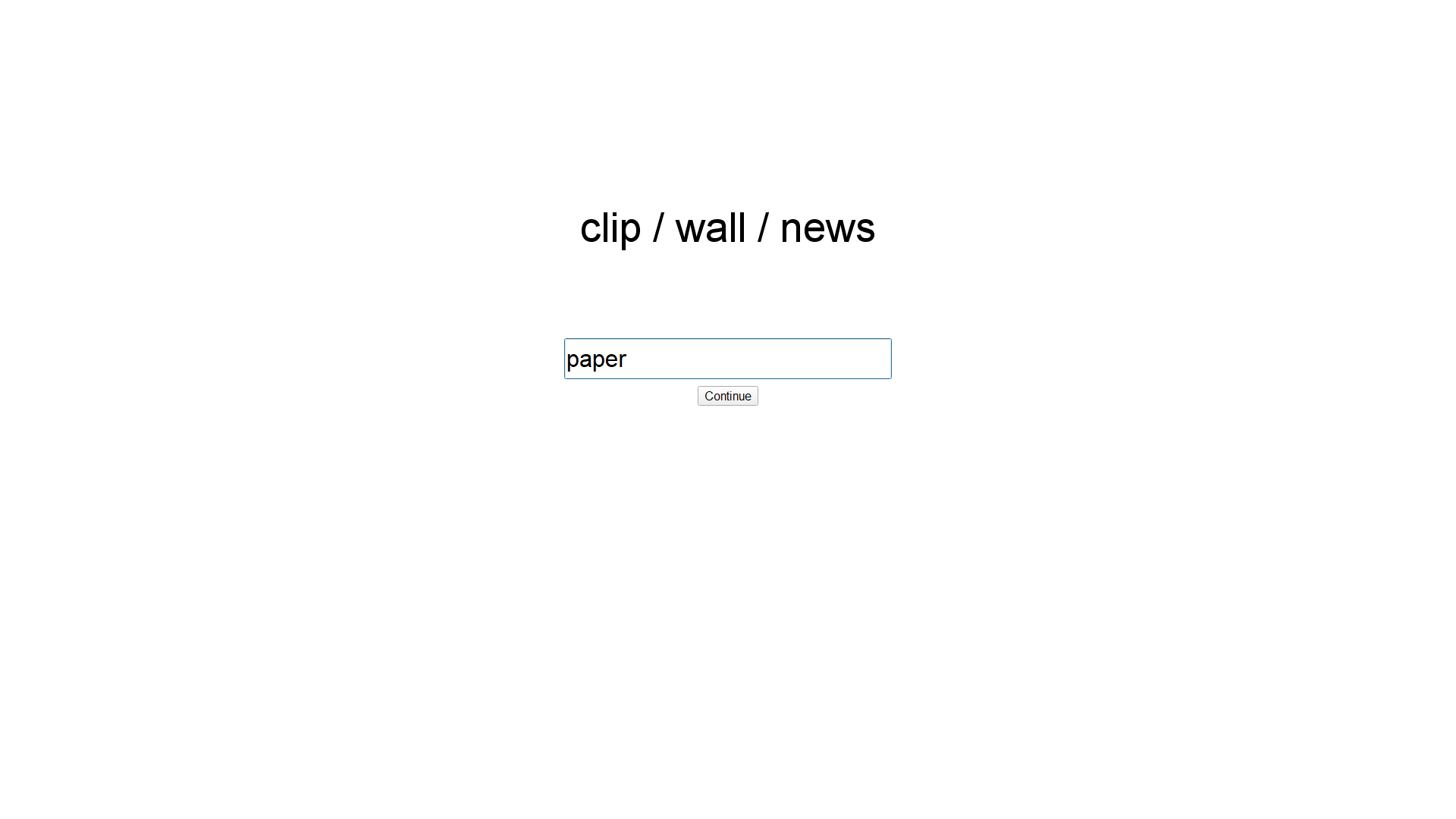


Figure D13: Sample Remote Associates Test trial

### Creative Foraging Game

The Creative Foraging Game (CFG) is a computerised task in which participants generate creative shapes by arranging ten identical squares (Hart et al., 2017). The initial condition is ten squares in a horizontal line. Participants can move the squares as they wish, but all squares must be connected to other square(s) by at least one shared edge. Participants will be asked to explore the space of possible shapes and save shapes they find interesting or creative into an in-game gallery. The gallery has no limit on the number of shapes. Participants will be told to play this game continuously for 12 minutes. After the 12 minutes, participants are asked to choose the five most creative shapes from their gallery. The exploration and exploitation optimality and originality scores (Hart et al., 2017) will be the outcome variables.


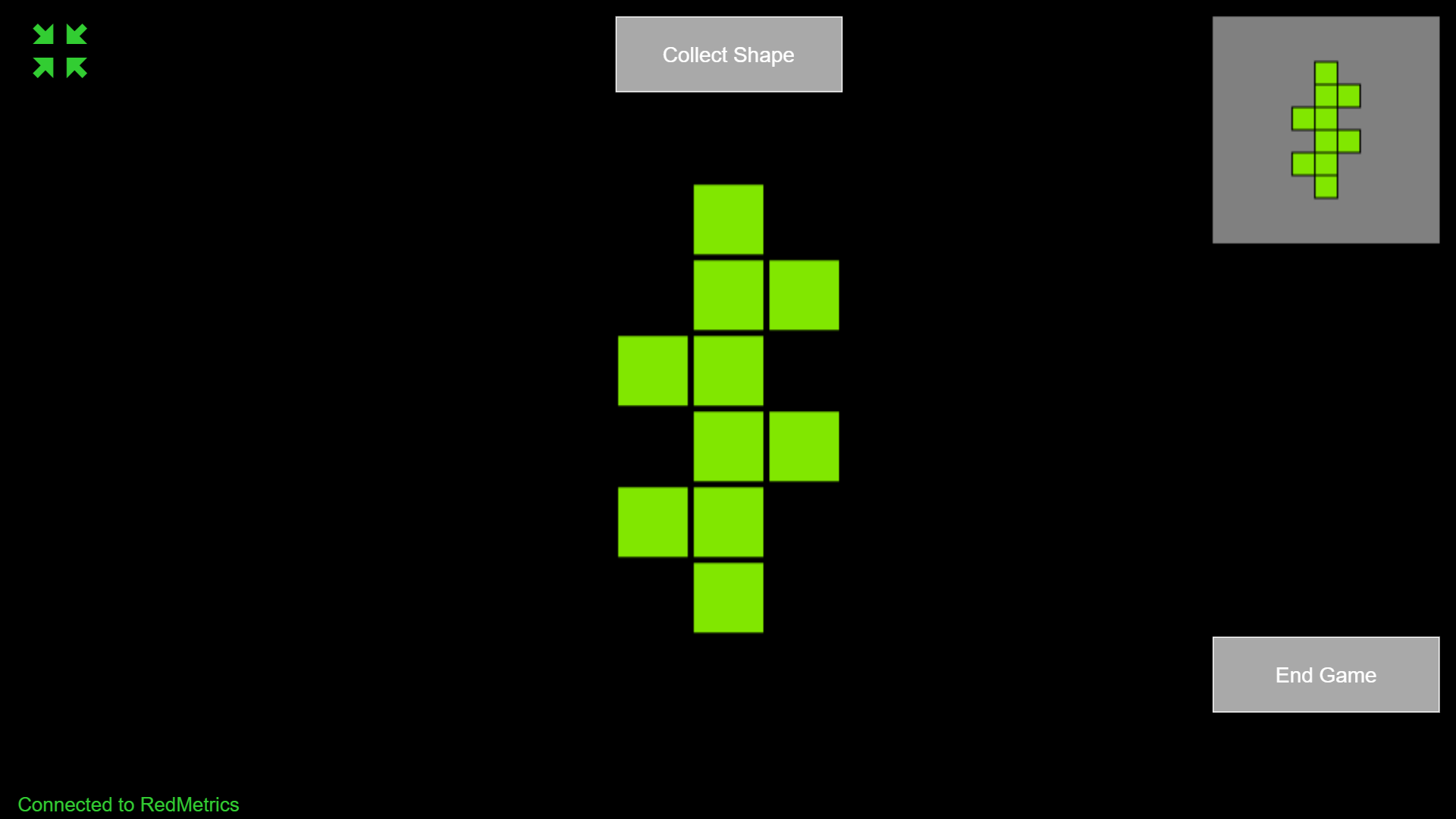


Figure D15: The CFG Task with the shape having been saved.

### Woodcock Johnson IV: Tests of Achievements

The Woodcock Johnson IV: Tests of Achievements is a comprehensive battery targeted at assessing areas of reading, writing, and maths in persons aged between 4 up to adult (Schrank & Wendling, 2018). Our study used the following subtests: Letter-Word Identification, Applied Problems, Passage Comprehension, Calculation, Sentence Reading Fluency, and Maths Facts Fluency. The outcome variables will be the total literacy scores and numeracy scores, detailed in the Woodcock Johnson IV scoring manual.

### Raven’s Progressive Matrices

To measure general intelligence, we use a computerised 18-item Raven’s Progressive Matrices (RPM) task (Sefcek et al., 2016), an aberrative version of the Raven's Advanced Progressive Matrices (John & Raven, 2003), on the Gorilla platform (www.gorilla.sc). Each RPM problem consists of visual geometric design with a missing piece. Participants are given eight options from which to choose to complete the missing component. The outcome variable is the number of correctly solved problems.

### Wechsler Abbreviated Scale of Intelligence II (WASI-II) Vocabulary and Block Design

Both Vocabulary and Block design are subtests from the Wechsler Abbreviated Scale of Intelligence II (Wechsler and Zhou, 2011). In the vocabulary task, participants will be asked to provide verbal definitions of target words, presented orally. In the block design task, participants will be asked to rearrange individual blocks that have different colours and patterns on different sides to make a target pattern. The outcome variables will be the vocabulary and block design scores, detailed in the WASI-II scoring manual.

### CANTAB One-Touch Stocking of Cambridge

Based on the Tower of Hanoi test, the CANTAB One-Touch Stocking of Cambridge (OTS) is a computerised test of executive function (Owen et al., 1990; Robbins et al., 1998). Two displays with three coloured balls are shown to the participant. The displays are designed to look like stacks of coloured balls held in stockings or socks strung from a beam. The goal is to reproduce the pattern in the upper display by moving the balls in the lower display. Participants must calculate the minimum number of moves the solutions require in their heads and respond by clicking the corresponding number. The number of problems solved on the first option and the mean latency (speed of response) to the first choice will be the outcome variables.

**
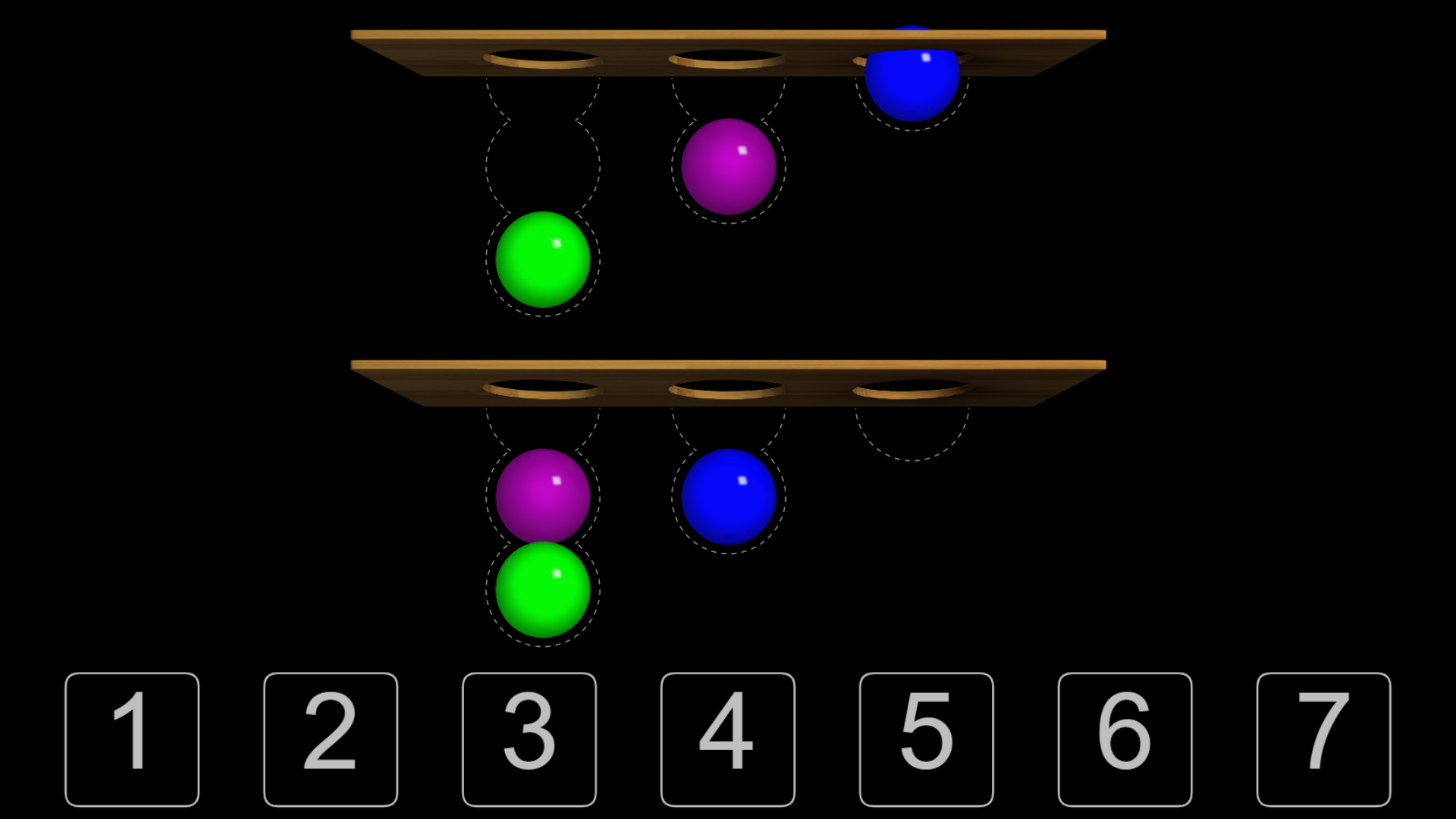
**

Figure D16: An OTS practice trial

## S5 Appendix E. Cognitive task battery testing order (Face-to-Face, Remote Guided Testing)

**Cognitive task battery testing order in Face-to-Face (F2F) testing mode**

|  | **Order 1** | **Order 2** | **Order 3** |
| --- | --- | --- | --- |
| **Day 1** | Structure Learning 1 | Task Set Switching | Spatial Working Memory |
|  | Probabilistic Reversal Learning | Wechsler Abbreviated Scale of Intelligence-Block Design | Intra-/Extra-Dimensional set shifting |
|  | Raven’s Progressive Matrices | Stop Signal Task | One-Touch Stocking of Cambridge |
|  | Wisconsin Card Sort Test | Structure Learning 1 | Creative Foraging Game |
|  | Spatial Working Memory | Probabilistic Reversal Learning | Trail Making Task |
|  | Intra-/Extra-Dimensional set shifting | Raven’s Progressive Matrices | Task Set Switching |
|  | One-Touch Stocking of Cambridge | Wisconsin Card Sort Test | Wechsler Abbreviated Scale of Intelligence-Block Design |
|  | Creative Foraging Game | Spatial Working Memory | Stop Signal Task |
|  | Trail Making Task | Intra-/Extra-Dimensional set shifting | Structure Learning 1 |
|  | Task Set Switching | One-Touch Stocking of Cambridge | Probabilistic Reversal Learning |
|  | Wechsler Abbreviated Scale of Intelligence-Block Design | Creative Foraging Game | Raven’s Progressive Matrices |
|  | Stop Signal Task | Trail Making Task | Wisconsin Card Sort Test |
| **Day 2** | Torrance Test of Creative Thinking-Figural | Structure Learning 2 | Remote Associates Test |
|  | Alternative Uses Task | Wechsler Abbreviated Scale of Intelligence-Vocabulary | Woodcock Johnson Letter-Word Identification |
|  | Verbal Fluency Test | Torrance Test of Creative Thinking-Figural | Stroop Task |
|  | Remote Associates Test | Alternative Uses Task | Backwards Digit Span |
|  | Woodcock Johnson Letter-Word Identification | Verbal Fluency Test | Structure Learning 2 |
|  | Stroop Task | Remote Associates Test | Wechsler Abbreviated Scale of Intelligence-Vocabulary |
|  | Backwards Digit Span | Woodcock Johnson Letter-Word Identification | Torrance Test of Creative Thinking-Figural |
|  | Structure Learning 2 | Stroop Task | Alternative Uses Task |
|  | Wechsler Abbreviated Scale of Intelligence-Vocabulary | Backwards Digit Span | Verbal Fluency Test |
| **Day 3** | Reading Span | Woodcock Johnson Passage Comprehension | Structure Learning 3 |
|  | Woodcock Johnson Applied Problems | Woodcock Johnson Calculation | Woodcock Johnson Passage Comprehension |
|  | Woodcock Johnson Maths Facts | Woodcock Johnson Sentence Reading | Woodcock Johnson Calculation |
|  | Structure Learning 23 | Reading Span | Woodcock Johnson Sentence Reading |
|  | Woodcock Johnson Passage Comprehension | Woodcock Johnson Applied Problems | Reading Span |
|  | Woodcock Johnson Calculation | Woodcock Johnson Maths Facts | Woodcock Johnson Applied Problems |
|  | Woodcock Johnson Sentence Reading | Structure Learning 23 | Woodcock Johnson Maths Facts |
|  | Survey | Survey | Survey |

**Cognitive task battery testing order in Remote Guided Testing (RGT) testing mode**

|  | **Order 1** | **Order 2** | **Order 3** |
| --- | --- | --- | --- |
| **Day 1** | Pre-Experiment Briefing & Check | | |
|  | Remote Associates Test | Structure Learning 1 | Reading Span |
|  | Woodcock Johnson Letter-Word Identification | Post Structure Learning Survey 1 | Woodcock Johnson Applied Problems |
|  | Alternative Uses Task | Reading Span | Creative Foraging Game |
|  | Structure Learning 1 | Woodcock Johnson Applied Problems | Remote Associates Test |
|  | Post Structure Learning Survey 1 | Creative Foraging Game | Woodcock Johnson Letter-Word Identification |
|  | Reading Span | Remote Associates Test | Alternative Uses Task |
|  | Woodcock Johnson Applied Problems | Woodcock Johnson Letter-Word Identification | Structure Learning 1 |
|  | Creative Foraging Game | Alternative Uses Task | Post Structure Learning Survey 1 |
| **Day 2** | Structure Learning 2 | Spatial Working Memory | Wechsler Abbreviated Scale of Intelligence-Vocabulary |
|  | Post Structure Learning Survey 2 | Intra-/Extra-Dimensional set shifting | Verbal Fluency Test |
|  | Probabilistic Reversal Learning | One-Touch Stocking of Cambridge | Woodcock Johnson Passage Comprehension |
|  | Wisconsin Card Sort Test | Trail Making Task | Raven’s Progressive Matrices |
|  | Spatial Working Memory | Wechsler Abbreviated Scale of Intelligence-Vocabulary | Structure Learning 2 |
|  | Intra-/Extra-Dimensional set shifting | Verbal Fluency Test | Post Structure Learning Survey 2 |
|  | One-Touch Stocking of Cambridge | Woodcock Johnson Passage Comprehension | Probabilistic Reversal Learning |
|  | Trail Making Task | Raven’s Progressive Matrices | Wisconsin Card Sort Test |
|  | Wechsler Abbreviated Scale of Intelligence-Vocabulary | Structure Learning 2 | Spatial Working Memory |
|  | Verbal Fluency Test | Post Structure Learning Survey 2 | Intra-/Extra-Dimensional set shifting |
|  | Woodcock Johnson Passage Comprehension | Probabilistic Reversal Learning | One-Touch Stocking of Cambridge |
|  | Raven’s Progressive Matrices | Wisconsin Card Sort Test | Trail Making Task |
| **Day 3** | Task Set Switching | Structure Learning 3 | Woodcock Johnson Maths Facts |
|  | Wechsler Abbreviated Scale of Intelligence-Block Design | Post Structure Learning Survey 3 | Torrance Test of Creative Thinking-Figural |
|  | Stop Signal Task | Woodcock Johnson Calculation | Stroop Task |
|  | Structure Learning 3 | Woodcock Johnson Sentence Reading | Task Set Switching |
|  | Post Structure Learning Survey 3 | Backwards Digit Span | Wechsler Abbreviated Scale of Intelligence-Block Design |
|  | Woodcock Johnson Calculation | Woodcock Johnson Maths Facts | Stop Signal Task |
|  | Woodcock Johnson Sentence Reading | Torrance Test of Creative Thinking-Figural | Structure Learning 3 |
|  | Backwards Digit Span | Stroop Task | Post Structure Learning Survey 3 |
|  | Woodcock Johnson Maths Facts | Task Set Switching | Woodcock Johnson Calculation |
|  | Torrance Test of Creative Thinking-Figural | Wechsler Abbreviated Scale of Intelligence-Block Design | Woodcock Johnson Sentence Reading |
|  | Stroop Task | Stop Signal Task | Backwards Digit Span |
|  | Survey | Survey | Survey |

#

## S6 Appendix F. Estimated Task Durations for Cognitive task battery

**Estimated Task Durations of the Cognitive Task Battery, including set-up for each task.**

| Task | Estimated Duration (Minutes) |
| --- | --- |
| Alternate Uses Task | 14 |
| Backwards Digit Span | 6 |
| Creative Foraging Game | 15 |
| Intra-/ Extra-Dimensional Set Shifting Task | 6 |
| One-Touch Stocking of Cambridge | 9 |
| Probabilistic Reversal Learning | 4 |
| Raven's Progressive Matrices | 14 |
| Reading Span | 10 |
| Remote Associates Test | 9 |
| Spatial Working Memory | 7 |
| Stop Signal Task | 12 |
| Stroop Task | 5 |
| Structure Learning Session 1 | 40 |
| Structure Learning Session 2 | 36 |
| Structure Learning Session 3 | 35 |
| Task Set Switching Test | 12 |
| Torrance Test of Creative Thinking - Figural | 33 |
| Trail-Making Test | 4 |
| Verbal Fluency Test | 8 |
| Wechsler Abbreviated Scale of Intelligence II - Block Design | 14 |
| Wechsler Abbreviated Scale of Intelligence II - Vocabulary | 17 |
| Wisconsin Card Sort Test | 4 |
| Woodcock Johnson Applied Problems | 21 |
| Woodcock Johnson Calculation | 14 |
| Woodcock Johnson Letter-Word Identification | 4 |
| Woodcock Johnson Maths Facts Fluency | 4 |
| Woodcock Johnson Passage Comprehension | 11 |
| Woodcock Johnson Sentence Reading Fluency | 5 |
| Total Task Duration | 373 minutes OR 6 hours 13 minutes |

## S7 Appendix G. Centre for Lifelong Learning and Individualised Cognition (CLIC) Phase 1 Consortium

Centre for Lifelong Learning and Individualised Cognition (CLIC) Phase 1 consortium is a collaborative research consortium dedicated to advancing knowledge and fostering innovation in the research of cognitive flexibility and its relationship with other executive functions and other key cognitive constructs like intelligence, creativity, and academic attainment. Our consortium brings together a diverse group of researchers from Nanyang Technological University, University of Cambridge, and other research institutions.

The following are the lists of

- CLIC Phase 1 Consortium Members, PIs and co-PIs
- CLIC Phase 1 Consortium Members, Admin and Research Staff
- CLIC External Collaborators
- CLIC Student Assistants

(Updated as of 26 June 2023)

## CLIC Phase 1 Consortium Members, PIs and co-PIs

| Name | Position | Institution |
| --- | --- | --- |
| Prof Zoe KOURTZI | PI | University of Cambridge |
| Prof Henriette HENDRIKS | PI | University of Cambridge |
| Prof. John SUCKLING | PI | University of Cambridge |
| Prof Annabel CHEN Shen-Hsing | PI | Nanyang Technological University |
| Assoc Prof Victoria LEONG | PI | Nanyang Technological University |
| Prof Balázs Zoltán GULYÁS | PI | Nanyang Technological University |
| Assoc Prof Georgios CHRISTOPOULOS | PI | Nanyang Technological University |
| Prof David HUNG | PI | Nanyang Technological University |
| Prof Trevor ROBBINS | Senior Scientific Advisor (PI) | University of Cambridge |
| Prof Barbara SAHAKIAN | Senior Scientific Advisor (PI) | University of Cambridge |
| Prof Michelle ELLEFSON | PI | University of Cambridge |
| Dr Chew Lee TEO | co-PI | Nanyang Technological University |
| Dr Sen Kee SEOW, Peter | co-PI | Nanyang Technological University |
| Assoc Prof Bobby CHEON* | PI | National Institutes of Health |

* indicates members who left CLIC as of the last update

## CLIC Phase 1 Consortium Members, Admin and Research Staff (non-PI)

| Name | Position | Institution |
| --- | --- | --- |
| Christine AYE Chan Myat | Senior Executive, Administration | Nanyang Technological University |
| Kastoori d/o KALAIVANAN* | Research Fellow | Nanyang Technological University |
| Xiaoqin CHENG* | Research Fellow | Nanyang Technological University |
| Ke TONG | Research Fellow | Nanyang Technological University |
| Ryutaro UCHIYAMA* | Research Fellow | Nanyang Technological University |
| Nastassja Lopes FISCHER | Research Fellow | Nanyang Technological University |
| Shengchuang FENG | Research Fellow | Nanyang Technological University |
| Nadhilla Velda MELIA | Research Fellow | Nanyang Technological University |
| Chia-Lun LIU | Research Fellow | Nanyang Technological University |
| Min LEE* | Research Associate | Nanyang Technological University |
| Boon Linn CHOO | Research Associate | Nanyang Technological University |
| Hui Shan YAP | Research Assistant | Nanyang Technological University |
| Jia Li TEO* | Research Assistant | Nanyang Technological University |
| Lisha Mohandas RAGHANI* | Research Assistant | Nanyang Technological University |
| Phillis Wei Li FU | Research Associate | Nanyang Technological University |
| Yan Fen TAN* | Research Assistant | Nanyang Technological University |
| Yuan Ni CHAN | Research Assistant | Nanyang Technological University |
| Irene MELANI* | Research Associate | Nanyang Technological University |
| Banani ANURAJ* | Senior Research Engineer | Nanyang Technological University |
| Jia Ying PEI* | Research Assistant | Nanyang Technological University |
| Li Ling LEE* | Research Assistant | Nanyang Technological University |
| Timothy LEE* | Research Associate | Nanyang Technological University |
| Yingqi CHUI* | Research Assistant | Nanyang Technological University |
| Sheng Hung CHUNG | Research Engineer | Nanyang Technological University |
| Kean Mun LEE | Research Assistant | Nanyang Technological University |
| Restria FAUZIANA | Research Associate | Nanyang Technological University |
| Natalie Philyra HOO Hui-Min | Research Assistant | Nanyang Technological University |
| Akshay ABRAHAM | Research Associate | Nanyang Technological University |
| Min HONG | Research Associate | Nanyang Technological University |
| Janet TAN Jia Yuan | Research Associate | Nanyang Technological University |
| Shamsul Azrin Bin JAMALUDDIN | Research Associate | Nanyang Technological University |
| Marisha Barth UBRANI | Research Assistant | Nanyang Technological University |
| FU Xinchen | Research Fellow | Nanyang Technological University |
| KOO Wei Ler | Research Assistant | Nanyang Technological University |
| Deepika SHUKLA | Research Fellow | Nanyang Technological University |
| Christelle LANGLEY | Affiliated Research Fellow | University of Cambridge |
| Chie TAKAHASHI | Affiliated Research Fellow | University of Cambridge |
| Aleya MARZUKI | Affiliated Research Fellow | Sunway University |

* indicates staff members who left CLIC as of the last update

##

## CLIC External Collaborators

| Name | Position | Institution |
| --- | --- | --- |
| Bobby CHEON | External Collaborator | National Institutes of Health |
| Yuval HART | External Collaborator | The Hebrew University of Jerusalem |
| Rudolf CARDINAL | External Collaborator | University of Cambridge |
| Jonathan ROISER | External Collaborator | University College London |
| WANG Rui | External Collaborator | Institute of Psychology, Chinese Academy of Sciences |
| Ryutaro UCHIYAMA | External Collaborator | University of Tübingen |
| Anahita TALWAR | External Collaborator | University College London |

##

## CLIC Student Assistants (SAs)

The SAs contributed to data collection in the CLIC Phase 1 adult characterisation study, adolescent characterisation study, and the adult intervention pilot study.

| Nurin Asyura Binte Rosle | Student Assistant | Nanyang Technological University |
| --- | --- | --- |
| Do You Jin | Student Assistant | Nanyang Technological University |
| Jayati RATHI | Student Assistant | Nanyang Technological University |
| Humaira Dwei Binte Aziz | Student Assistant | Nanyang Technological University |
| KNG Jia Hao | Student Assistant | Nanyang Technological University |
| FOONG Wai Teng, Melissa | Student Assistant | Nanyang Technological University |
| Benjamin SOON Jien Weng | Student Assistant | Nanyang Technological University |
| Anthony HEW Joon Kit | Student Assistant | Nanyang Technological University |
| Jocelyn TOH Sze Lyn | Student Assistant | Nanyang Technological University |
| LEE Yan Faye | Student Assistant | Nanyang Technological University |
| LI Yujin | Student Assistant | Nanyang Technological University |
| LEE Yu Ming | Student Assistant | Nanyang Technological University |
| SAWANT Sachi Vilas | Student Assistant | Nanyang Technological University |
| SIM En Qi | Student Assistant | Nanyang Technological University |
| WONG Jia Wen | Student Assistant | Nanyang Technological University |
| ONG Jodie | Student Assistant | Nanyang Technological University |
| Goh Ling Yi | Student Assistant | Nanyang Technological University |
| YEO Zi Yi, Rina | Student Assistant | Nanyang Technological University |
| Bernice Ho Tze Yan | Student Assistant | Nanyang Technological University |
| Adarsh Richelle | Student Assistant | Nanyang Technological University |
| Amanda Chia | Student Assistant | Nanyang Technological University |
| Rithu Ann Mathew | Student Assistant | Nanyang Technological University |
| Aabha Hattangadi | Student Assistant | Nanyang Technological University |
| Cui Chen | Student Assistant | Nanyang Technological University |
| Chan Tong Ling | Student Assistant | Nanyang Technological University |
| Dylann Lee Yong Han | Student Assistant | Nanyang Technological University |
| Wang Jie Qi | Student Assistant | Nanyang Technological University |
| Loh Sher Leng | Student Assistant | Nanyang Technological University |
| GOH Pei Swhen | Student Assistant | Nanyang Technological University |
| Ciera WONG | Student Assistant | Nanyang Technological University |
| Neha Vijayanand | Student Assistant | Nanyang Technological University |
| Annika TANG Siting | Student Assistant | Nanyang Technological University |
| CHING Kai Ling | Student Assistant | Nanyang Technological University |
| Katarina Koesoemo | Student Assistant | Nanyang Technological University |
| Mercado Alyzza Francesca Marmita | Student Assistant | Nanyang Technological University |
| Nashua Nizar Baledram | Student Assistant | Nanyang Technological University |
| Angel Lee Jia Ying | Student Assistant | Nanyang Technological University |
| Jan Paolo Macapinlac Balagtas | Student Assistant | Nanyang Technological University |
| Lau Su Ching | Student Assistant | Nanyang Technological University |
| Cassia Pang Xin Ying | Student Assistant | Nanyang Technological University |
| Grace Kon Xin Xuan | Student Assistant | Nanyang Technological University |
| Kan E Shan Erica | Student Assistant | Nanyang Technological University |
| Lee Hui Min Sandra | Student Assistant | Nanyang Technological University |
| Sherlyn Lee Yee Ying | Student Assistant | Nanyang Technological University |
| Teh Zhi Jing | Student Assistant | Nanyang Technological University |
| Jacques Ting Shu Rui | Student Assistant | Nanyang Technological University |
| Ng Wei Zhou | Student Assistant | Nanyang Technological University |
| Edward Say Jun De | Student Assistant | Nanyang Technological University |
| Celine Chan Shu Ying | Student Assistant | Nanyang Technological University |
| Lim Shee-Ann | Student Assistant | Nanyang Technological University |
| Ashley Choo Yan Zeng | Student Assistant | Nanyang Technological University |
| Chan Shu Ting, Carol | Student Assistant | Nanyang Technological University |
| Wang Ziyi | Student Assistant | Nanyang Technological University |
| Amanda Tan | Student Assistant | Nanyang Technological University |
| Lucas Tan Rui Yu | Student Assistant | Nanyang Technological University |
| Ng Kai Li | Student Assistant | Nanyang Technological University |
| Tan Rong Hui | Student Assistant | Nanyang Technological University |
| Michelle Soh Wei Ting (Su Weiting) | Student Assistant | Nanyang Technological University |
| Renee Kuek Yan Ran | Student Assistant | Nanyang Technological University |
| Katarina Koesoemo | Student Assistant | Nanyang Technological University |
